# Functional Nitrogenase Cofactor Maturase NifB in Mitochondria and Chloroplasts of *Nicotiana benthamiana*

**DOI:** 10.1128/mbio.00268-22

**Published:** 2022-06-13

**Authors:** Xi Jiang, Diana Coroian, Emma Barahona, Carlos Echavarri-Erasun, Rocío Castellanos-Rueda, Álvaro Eseverri, Jose A. Aznar-Moreno, Stefan Burén, Luis M. Rubio

**Affiliations:** a Centro de Biotecnología y Genómica de Plantas, Universidad Politécnica de Madrid, Instituto Nacional de Investigación y Tecnología Agraria y Alimentaria, Pozuelo de Alarcón, Madrid, Spain; b Departamento de Biotecnología-Biología Vegetal, Escuela Técnica Superior de Ingeniería Agronómica, Alimentaria y de Biosistemas, Universidad Politécnica de Madrid, Madrid, Spain; University of California, Berkeley

**Keywords:** FeMo-cofactor, metalloproteins, *nif*, nitrogen fixation, synthetic biology

## Abstract

Engineering plants to synthesize nitrogenase and assimilate atmospheric N_2_ will reduce crop dependency on industrial N fertilizers. This technology can be achieved by expressing prokaryotic nitrogen fixation gene products for the assembly of a functional nitrogenase in plants. NifB is a critical nitrogenase component since it catalyzes the first committed step in the biosynthesis of all types of nitrogenase active-site cofactors. Here, we used a library of 30 distinct *nifB* sequences originating from different phyla and ecological niches to restore diazotrophic growth of an Azotobacter vinelandii *nifB* mutant. Twenty of these variants rescued the *nifB* mutant phenotype despite their phylogenetic distance to A. vinelandii. Because multiple protein interactions are required in the iron-molybdenum cofactor (FeMo-co) biosynthetic pathway, the maturation of nitrogenase in a heterologous host can be divided in independent modules containing interacting proteins that function together to produce a specific intermediate. Therefore, *nifB* functional modules composed of a *nifB* variant, together with the A. vinelandii NifS and NifU proteins (for biosynthesis of NifB [Fe_4_S_4_] clusters) and the FdxN ferredoxin (for NifB function), were expressed in Nicotiana benthamiana chloroplasts and mitochondria. Three archaeal NifB proteins accumulated at high levels in soluble fractions of chloroplasts (Methanosarcina acetivorans and Methanocaldococcus infernus) or mitochondria (*M. infernus* and Methanothermobacter thermautotrophicus). These NifB proteins were shown to accept [Fe_4_S_4_] clusters from NifU and were functional in FeMo-co synthesis *in vitro*. The accumulation of significant levels of soluble and functional NifB proteins in chloroplasts and mitochondria is critical to engineering biological nitrogen fixation in plants.

## INTRODUCTION

More than half of the food currently consumed in the world is produced using synthetic nitrogen (N) fertilizers, an industry that consumes about 1% of the global energy supply (1, 2). Unfortunately, crops only assimilate about 30% of the applied N fertilizer (3), while the rest of it leaches from the soil constituting a major source of pollution with tremendous negative environmental consequences (4, 5). Because plants cannot fix their own nitrogen, introducing biological nitrogen fixation (BNF) into cereals is necessary to reduce our dependency on N fertilizers and preserve the environment while sustaining productivity (6, 7). BNF is the conversion of inert atmospheric dinitrogen gas (N_2_) into nitrogen-reactive ammonia (NH_3_), a reaction catalyzed by the nitrogenase enzyme that is present only in diazotrophic bacteria and archaea (8).

One approach to obtain nitrogen-fixing cereal crops is to transfer the prokaryotic nitrogen fixation (*nif*) genes to the plant (7). Nitrogenases are enzyme complexes of two interacting components, a catalytic component called dinitrogenase and an electron donor component called dinitrogenase reductase (9). The nitrogenase reaction involves a minimum of eight cycles of electron transfer and component association/dissociation driven by ATP hydrolysis (10). There are three types of nitrogenases: molybdenum (Mo), vanadium (V), and iron-only (Fe) nitrogenases. They are genetically distinct but functionally and structurally similar, with some differences in subunit and cofactor composition (11). Mo-nitrogenase is the most widespread, the most efficient in N_2_ reduction, and the best characterized (12). The dinitrogenase reductase component of Mo-nitrogenase is a homodimer of the *nifH* gene product that coordinates a low potential redox-active [Fe_4_S_4_] cluster and is responsible for Mg·ATP hydrolysis. The catalytic component (NifDK) corresponds to an α_2_β_2_-tetramer with two unique metalloclusters per αβ-dimer: iron-molybdenum cofactor (FeMo-co) and P-cluster. The [Fe_8_S_7_] P-cluster is responsible for the successive electron transfers from the [Fe_4_S_4_] cluster of NifH to the [MoFe_7_S_9_C-*R*-homocitrate] FeMo-co, the enzyme catalytic cofactor (13). These three metalloclusters are irreversibly destroyed by O_2_.

FeMo-co biosynthesis is initiated by the NifS-directed assembly of [Fe_4_S_4_] cluster units at NifU, which are then transferred to NifB (14). NifB is an *S*-adenosyl-l-methionine (SAM) radical enzyme that converts two [Fe_4_S_4_] cluster units into an [Fe_8_S_9_C] cluster called NifB-co (15–18). A radical-generated central carbide, originating from SAM (19), and a ninth sulfide are also incorporated during NifB reaction (20). NifB-co is then transferred, either directly or via NifX, to the NifEN scaffold, where it is converted into FeMo-co in association with NifH (21–23). Mo and homocitrate for FeMo-co are donated by NifQ and NifV, respectively (24). Importantly, NifB is also required for the biosynthesis of the active site cofactors of the V and Fe-only nitrogenases (17, 25), which arguably makes it the most important protein in global nitrogen fixation.

About 70% of NifB sequences identified in databases contain an N-terminal SAM-radical region and a C-terminal NifX-like region (26, 27). Expression of two-domain NifB proteins from model diazotrophs Azotobacter vinelandii and Klebsiella oxytoca in heterologous systems has proven difficult, often resulting in insoluble protein accumulation (15, 28, 29). However, functional NifB proteins consisting of a standalone SAM-radical domain, found in methanogens and other prokaryotes (18, 26, 30), could be expressed in yeast. When a library of 30 NifB variants of different phylogenetic origins was expressed and targeted to yeast mitochondria, six variants accumulated as soluble proteins and two were isolated and showed *in vitro* functionality (31).

Here, we have expressed 30 distinct *nifB* genes in A. vinelandii and observed that 20 could replace the function of the native *nifB* gene. The origin of these 20 genes and their protein sequences showed low sequence similarity to that of A. vinelandii, demonstrating that the NifB product (NifB-co) itself is the determining factor for cluster delivery and suggesting that engineering of nitrogenase in heterologous hosts can be divided in independent modules in which the end product from one module (e.g., Fe-S cluster biosynthesis, in this case NifB-co) can be transferred to another module (e.g., the corresponding apo-protein, in this case NifEN), although the components within each module originate from different species. We then expressed and targeted the 30 NifB proteins to mitochondria or chloroplasts in Nicotiana benthamiana in two parallel screening and selection procedures. Three NifB proteins from methanogens were found soluble and accumulated abundantly either in chloroplasts or mitochondria. Importantly, selected NifB proteins supported *in vitro* FeMo-co synthesis using [Fe_4_S_4_] cluster substrates provided by the physiological donor NifU, proving that these identified variants were isolated as functional proteins from both plant organelles.

## RESULTS

### *In vivo* functionality of NifB proteins.

**FIG 1:**
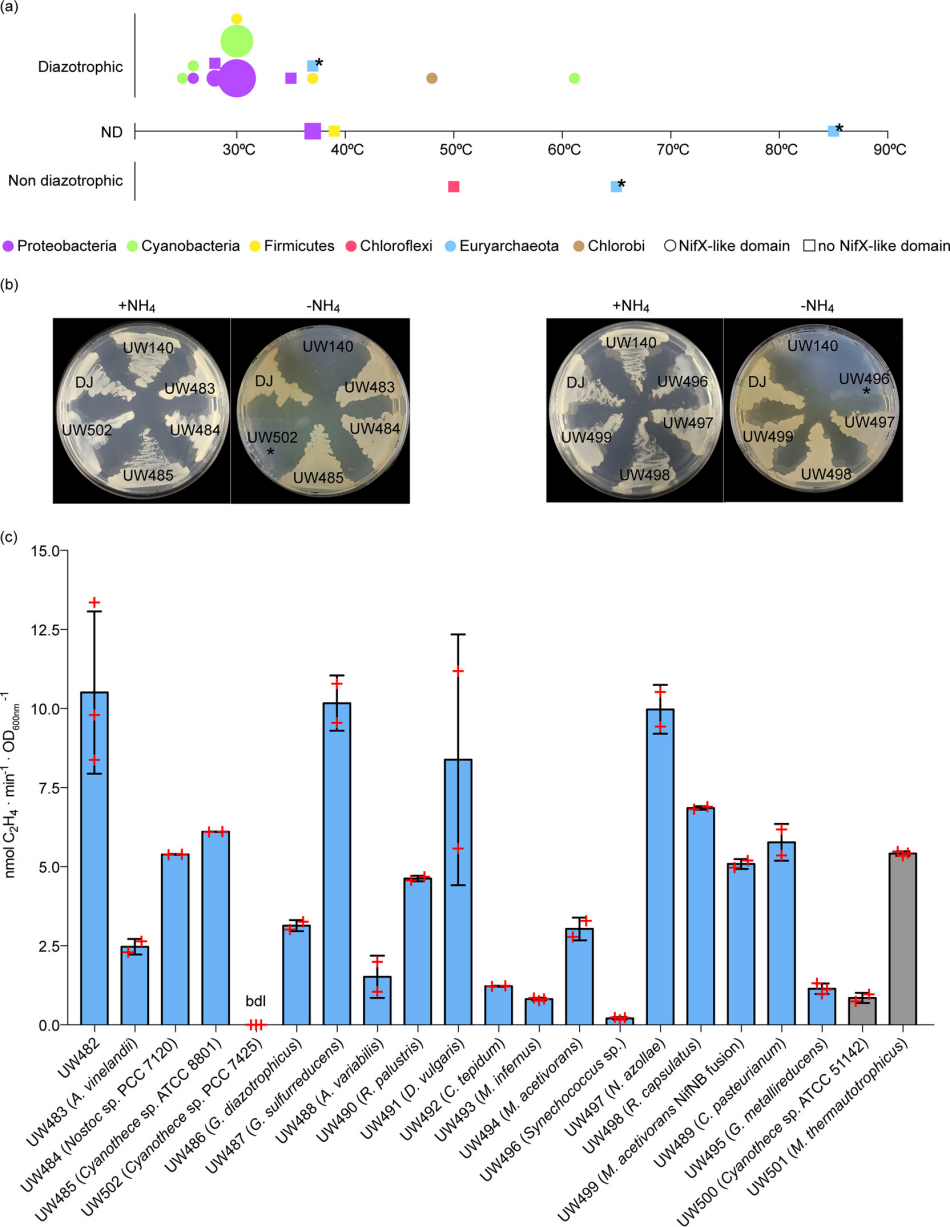
Representation of the NifB library and *in vivo* complementation of A. vinelandii strain UW140 (Δ*nifB*) with TS-NifB variants. (a) Plot of the organisms from where the NifB protein variants were selected. Four features are indicated: (i) growth temperature (x axis); (ii) nitrogen fixing capacity (y axis), where confirmed diazotrophs are above the x axis, organisms with no published data regarding N-fixing capacity are in line with the x axis, and nondiazotrophs are below the x axis; (iii) phylum, represented by color (see color scheme at the bottom of the panel); and (iv) presence (circle) or absence (square) of the NifX-like domain at each NifB protein. The size of each symbol corresponds to the number of representatives sharing same features. Black stars indicate NifB variants shown soluble in tobacco organelles. (b) Growth comparison of A. vinelandii UW140 (Δ*nifB*) strain complemented with some *ts-nifB* on plates with ammonium (+NH_4_) or N_2_ (-NH_4_) as sole nitrogen source. A. vinelandii DJ is the wild-type strain. The stars indicate complemented strains with phenotype of slow diazotrophic growth on solid medium. (c) Acetylene reduction activity measured at 18 h (blue bars) or 42 h (grey bars) after nitrogen source removal from the media. Red crosses represent individual biological replicates. ARA activities are presented as nanomoles of ethylene produced per minute and the OD_600_. UW482 is the wild-type strain DJ transformed with the parental empty vector pN2SB51. *Cyanothece* sp. strain PCC 7425 (*nifB*) restored the Nif^+^ phenotype, but no acetylene reduction activity could be measured at 18 h or 42 h (bdl, below detection limit). The activity of the A. vinelandii DJ wild type was 21.1 ± 0.2 U. Error bars indicate means ± the standard deviations (SD; *n* ≥ 2 biological replicates).

To assess *in vivo* activity and cross-compatibility of NifB variants with the rest of the A. vinelandii proteins required for FeMo-co synthesis, each one of the 30 *nifB* genes (Fig. 1a; see also Tables S1 and S2 in the supplemental material) was individually used for genetic complementation experiments of the A. vinelandii UW140 (Δ*nifB*) Nif^–^ strain (15). A sequence encoding the TwinStrep tag (TS-tag) was placed at the 5′ end of the *nifB* genes to allow for NifB variants detection. Of the 30 NifB variants, 18 restored UW140 growth on N_2_ and supported various levels of nitrogenase activity, as measured by an acetylene reduction assay (ARA) (Fig. 1b and c). Two NifB variants (*Cyanothece* sp. strain PCC 7425 and *Synechococcus* sp.) did not support significant acetylene reduction under the conditions tested but restored diazotrophic growth when plates were incubated for longer time periods. Detection of the NifB protein variants by immunoblotting was unsuccessful when probing for the TS-tag, similarly to what we observed when expressing TS-NifH*^Ht^* in A. vinelandii (32). However, the NifB*^Av^* protein could be detected in strain UW483 (see Table S1) using a polyclonal antibody raised against the A. vinelandii NifB protein (see Fig. S1 in Text S1 in the supplemental material).

10.1128/mbio.00268-22.1 TEXT S1 Supplemental Materials and Methods and Fig. S1 to S7. Download Text S1, PDF file, 4.7 MB. Copyright © 2022 Jiang et al. 2022 Jiang et al. https://creativecommons.org/licenses/by/4.0/ This content is distributed under the terms of the Creative Commons Attribution 4.0 International license.

10.1128/mbio.00268-22.2 TABLE S1 List of vectors used to complement A. vinelandii UW140 (Δ*nifB*). Download Table S1, PDF file, 0.1 MB. Copyright © 2022 Jiang et al. 2022 Jiang et al. https://creativecommons.org/licenses/by/4.0/ This content is distributed under the terms of the Creative Commons Attribution 4.0 International license.

10.1128/mbio.00268-22.3 TABLE S2 Information about the NifB variants used in the library screening. Download Table S2, XLSX file, 0.05 MB. Copyright © 2022 Jiang et al. 2022 Jiang et al. https://creativecommons.org/licenses/by/4.0/ This content is distributed under the terms of the Creative Commons Attribution 4.0 International license.

About 90% (7/8) of the NifB variants from the *Cyanobacteria* phylum, 100% (4/4) from *Euryarchaeota*, 33% (1/3) from *Firmicutes*, 100% (1/1) from *Chlorobi*, 0% (0/1) *Chloroflexi*, and 54% (7/13) from *Proteobacteria* reverted the Nif^–^ phenotype of UW140. There was no correlation between complementation ability and NifB protein sequences or phylogenic distances of the organisms from where the NifB proteins originated to A. vinelandii (see Fig. S2 in Text S1), suggesting that NifB-co delivery from NifB to either NifX or NifEN is not mediated via discriminating NifB-NifX/NifEN interactions but rather that the NifB-co structure itself is the determining factor for specific cluster delivery from NifB to its subsequent partners.

### NifB variant solubility in mitochondria and chloroplasts of tobacco.

**FIG 2:**
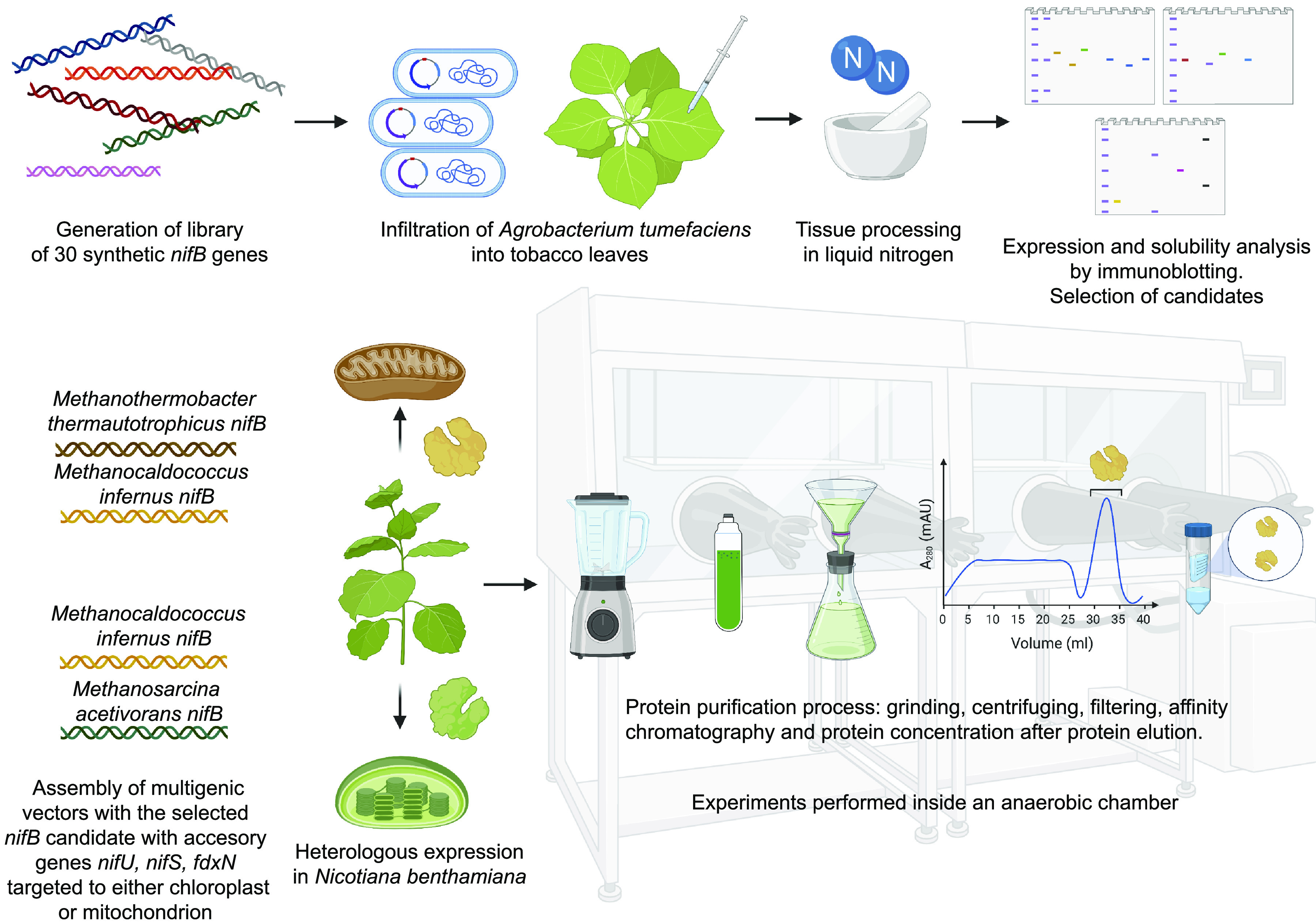
Experimental design and execution of tobacco NifB libraries. Two libraries of 30 *ts*-*nifB* genes with targeting sequences for chloroplasts or mitochondria were constructed and cloned in binary plasmids. The TS-NifB proteins were transiently expressed in A. tumefaciens-infiltrated tobacco leaves together with NifU, NifS, FdxN, GFP, and p19. NifB expression (i.e., NifB present in total protein extracts) and solubility (i.e., NifB present in soluble fraction of protein extracts) were determined by immunoblot analysis of the protein extracted from the homogenized leaf tissue. The genes encoding soluble NifB variants (for mitochondria and chloroplasts, respectively) were assembled with *nifU*, *nifS*, and *fdxN* in multigenic vectors. Large-scale A. tumefaciens infiltrations were performed to obtain sufficient leaf material for NifB isolation by STAC.

Each NifB variant, together with NifU, NifS, and FdxN, was targeted to either chloroplasts or mitochondria of N. benthamiana leaves (Fig. 2). The library directing Nif proteins to mitochondria was constructed with targeting signals COX4 (for NifB) and SU9 (for NifU, NifS, and FdxN) (28, 33, 34), whereas the library targeting the proteins to chloroplasts used the SSU targeting signal (35). Protein targeting to the mitochondria in tobacco using these SU9 and COX4 sequences have previously been verified using confocal microscopy (28) but also in rice (36). Successful translocation into the tobacco chloroplast using SSU was shown using green fluorescent protein (GFP) (see Fig. S3 in Text S1). The TS-tag was placed between targeting signals and NifB for variant detection, quantification, and isolation by Strep-Tactin affinity chromatographic purification (STAC). A similar construct design was previously shown to be efficient for NifH purification from tobacco mitochondria and chloroplasts (32, 37). For Agrobacterium tumefaciens-mediated transient expression in leaves, the *nifB* genes were cloned into binary vectors together with a *GFP* transcriptional unit (see Table S3). Expression of cytoplasmic GFP was used to verify successful A. tumefaciens infiltration and normalize NifB expression. Leaves were simultaneously infiltrated with two additional A. tumefaciens strains: one containing a binary plasmid for expression of mitochondrion or chloroplast targeted NifU, NifS, and FdxN, and another for expression of the p19 RNA silencing suppressor (38).

10.1128/mbio.00268-22.4 TABLE S3 Plant binary vectors used in this study. Download Table S3, PDF file, 0.1 MB. Copyright © 2022 Jiang et al. 2022 Jiang et al. https://creativecommons.org/licenses/by/4.0/ This content is distributed under the terms of the Creative Commons Attribution 4.0 International license.

**FIG 3:**
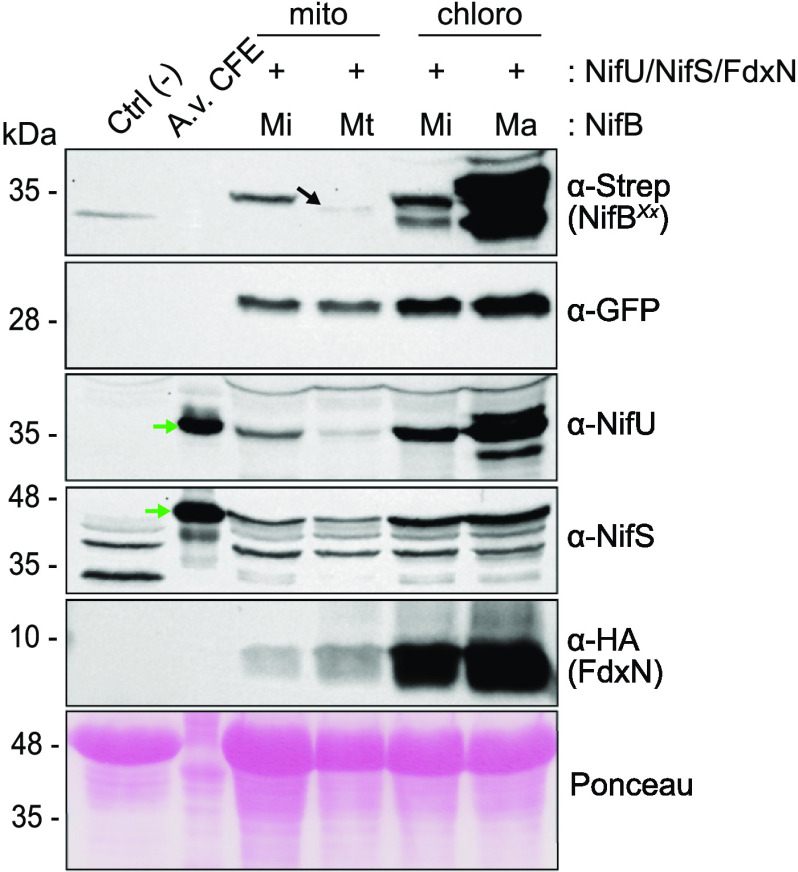
Expression of soluble NifB candidates in mitochondria and chloroplasts. Immunoblot analysis of soluble N. benthamiana protein extracts with antibodies against the TS-tag (α-Strep, for NifB detection), GFP, NifU, NifS, and the HA tag (for FdxN detection). “Ctrl (–)” indicates total protein extract from nontransformed tobacco leaves. “A.v. CFE” is A. vinelandii soluble protein extract used as a control for the migration of NifU and NifS. The black arrow indicates NifB*^Mt^*; the green arrows indicate NifU and NifS.

Most NifB variants were well expressed from both libraries (see Fig. S4 in Text S1), but only few accumulated as soluble proteins (see Fig. S5 in Text S1). For the mitochondrion library, those originating from Methanocaldococcus infernus (NifB*^Mi^*), Methanosarcina acetivorans (NifB*^Ma^*), and Methanothermobacter thermautotrophicus (NifB*^Mt^*) were soluble (Fig. 3). The SDS-PAGE migration pattern of NifB*^Ma^* suggested extensive protein degradation, and this variant was excluded from further work (see Fig. S5 in Text S1). For the chloroplast library, NifB*^Mi^* and NifB*^Ma^* were soluble. A faster-migrating form of NifB*^Ma^* was present, but much less prominently than in mitochondria, suggesting specific proteolytic cleavage during mitochondrial import as previously reported for NifD (39, 40). Since this faster-migrating form of NifB*^Ma^* was detected using the Strep-tag antibody, the protein was likely truncated in the C terminus.

### Purification of NifB proteins from mitochondria and chloroplasts of tobacco leaf cells.

The four selected TS-tagged NifB proteins were expressed at larger scale in tobacco leaves (NifB*^Mi^* and NifB*^Mt^* targeted to mitochondria, and NifB*^Mi^* and NifB*^Ma^* targeted to chloroplasts), and isolated for functional analysis using STAC (Fig. 2). To ensure efficient and stoichiometric coexpression of the accessory proteins, multigenic vectors comprising *ts-nifB*, *nifU*, *nifS*, *fdxN-HA*, and *GFP* with the gene silencing suppressor *p19* were constructed using MoClo (41) (see Table S3). NifB, NifU, NifS, and FdxN were targeted to mitochondria or chloroplasts, while GFP and p19 were expressed in the cytosol.

**FIG 4:**
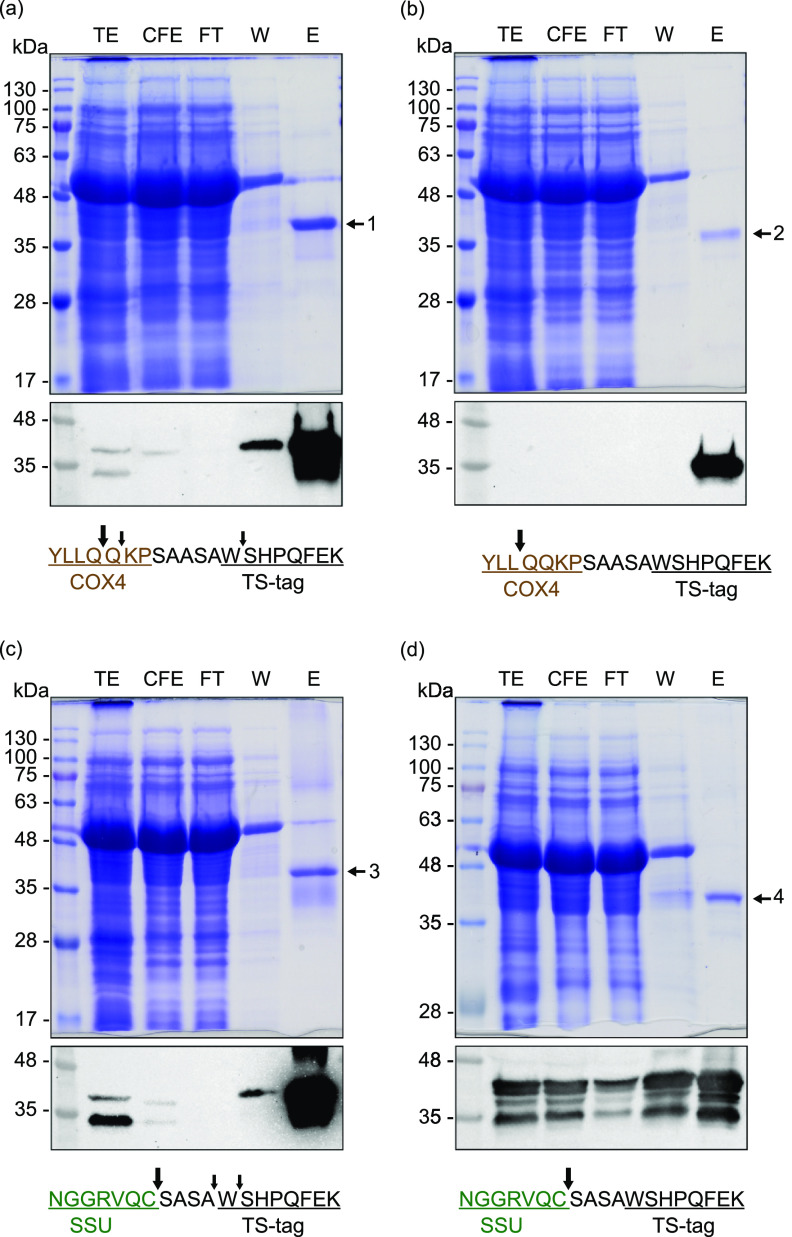
Purification of soluble NifB proteins from tobacco leaf mitochondria and chloroplasts. Analysis of STAC purification fractions was performed for mitochondrial NifB*^Mi^* (a), mitochondrial NifB*^Mt^* (b), chloroplast NifB*^Mi^* (c), and chloroplast NifB*^Ma^* (d). Each panel shows the analysis of a representative purification process using Coomassie blue-stained SDS-PAGE and immunoblot analysis with Streptactin-HRP to detect TS-NifB variants. TE, total extract; CFE, cell extract; FT, flowthrough fraction; W, wash fraction; E, elution fraction. Bands 1 to 4 were analyzed by peptide mass fingerprinting and identified as NifB*^Mi^* (D5VRM1, 55% sequence coverage), NifB*^Mt^* (O27899, 70% sequence coverage), NifB*^Mi^* (D5VRM1, 27% sequence coverage), and NifB*^Ma^* (Q8TIF7, 41% sequence coverage), respectively. Significance peptide mass fingerprint thresholds, *P* < 0.05. The processing sites for COX4 (mitochondria) and SSU (chloroplast) targeting peptides, as determined by N-terminal sequencing, are shown below each panel. Cleavage sites are indicated by arrows. The dominant processing site is indicated by a larger arrow.

A minimum of three STAC purifications were performed for each NifB variant from tobacco leaves harvested at the end of the dark period 4 days following A. tumefaciens infiltration. Leave tissue homogenization and protein purification were performed under anaerobic conditions inside a glove box. Representative immunoblots and Coomassie blue-stained gels of samples collected throughout the purification process are shown in Fig. 4. The elution fractions contained putative NifB and contaminating proteins. The predominant band from each elution fraction was excised and subjected to peptide fingerprinting to confirm NifB identity (Fig. 4). Amino-terminal sequencing confirmed correct transit peptide processing of NifB proteins in mitochondria and chloroplasts (Fig. 4). NifB in final preparations was quantified by band densitometric analysis of Coomassie-stained SDS gels compared to calibration standards made with pure TS-NifB*^Mi^* from Saccharomyces cerevisiae (see Fig. S6 in Text S1). Purification yields for the NifB proteins are listed in Table 1. The smallest amount was obtained for mitochondria NifB*^Mt^* and the highest for chloroplast NifB*^Ma^*, although there was variation between individual experiments. NifB*^Mi^* was isolated to similar levels independently of the target organelle. In general, tobacco organelles yielded 20-fold less NifB protein than the amount reported for NifB*^Mi^* from yeast mitochondria (31).

**TABLE 1:** Yields of NifB variants isolated from tobacco leaves

Organelle	Protein	Mean yield (μg/g of leaves) ± SD	No. of biological replicates
Mitochondrion	NifB*^Mi^*	3.31 ± 1.43	4
	NifB*^Mt^*	0.53 ± 0.25	4
Chloroplast	NifB*^Mi^*	4.17 ± 1.12	3
	NifB*^Ma^*	7.12 ± 5.45	3

### FeMo-co synthesis using NifB isolated from mitochondria and chloroplasts.

NifB activity was assayed by the NifB-dependent *in vitro* assay of FeMo-co synthesis and insertion into the P-cluster containing (but FeMo-co free) apo-NifDK (24). Activated NifDK was then measured by the ARA. This assay requires NifB, NifEN, NifH, and apo-NifDK in their purest forms along with SAM, molybdate, and *R*-homocitrate (42). NifB must contain its full complement of three [Fe_4_S_4_] clusters (the catalytic RS cluster and the substrate K1 and K2 clusters) to render the NifB-co functional for FeMo-co synthesis. However, a very small proportion of NifB is normally isolated in this state primed for catalysis, and its [Fe_4_S_4_] clusters are first reconstituted *in vitro* by incubation with ferrous iron and sulfide in the presence of dithiothreitol (DTT) (42).

In this study, we aimed at analyzing functionality and quality of NifB isolated from tobacco organelles. Since it was possible that the standard chemical reconstitution of NifB clusters could result in artificially high NifB activities masking defects such as damaged clusters, we used instead holo-NifU (i.e., NifU loaded with [Fe_4_S_4_] clusters) to reconstitute the full complement of NifB [Fe_4_S_4_] clusters (see Materials and Methods for details). NifU is the *in vivo* donor of [Fe_4_S_4_] clusters to NifB (14). After FeMo-co synthesis, activation of NifDK was measured by the ARA according to standard methods (see Fig. S7 in Text S1 in the supplemental material).

**FIG 5:**
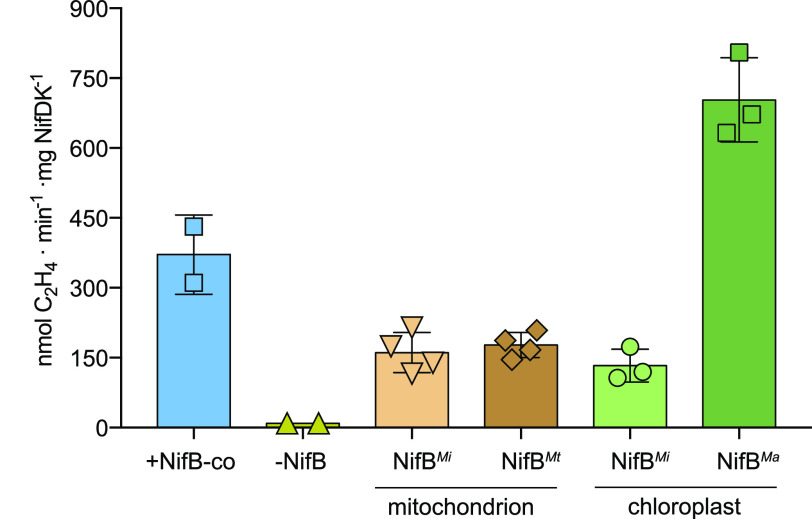
FeMo-co synthesis and activation of apo-NifDK using NifB purified from tobacco. NifB-dependent *in vitro* FeMo-co synthesis and apo-NifDK activation using tobacco-purified NifB variants from mitochondria and chloroplasts assayed with purified A. vinelandii Nif protein components. A positive control for FeMo-co synthesis and apo-NifDK activation was performed by replacing NifB by pure NifB-co. A 20:1 molar ratio of NifH to NifDK was used in the ARA. *n* = 2 technical replicates (+NifB-co and –NifB-co), *n* = 4 biological replicates (NifB*^Mi^* and NifB*^Mt^* isolated from mitochondria), *n* = 3 biological replicates (NifB*^Mi^* and NifB*^Ma^* isolated from chloroplasts). Activity is represented as nanomoles of ethylene produced per minute and milligram of NifDK. Error bars represent means ± the SD.

Tobacco-purified NifB proteins proved to be functional (Fig. 5). NifB*^Mi^* activities were similar regardless of being expressed in mitochondria or chloroplasts. The activity of NifB*^Mt^* expressed in mitochondria was similar to NifB*^Mi^*, while NifB*^Ma^* expressed in chloroplasts exhibited 5-fold higher activity than NifB*^Mi^* and NifB*^Mt^*. In any case, the activities of tobacco-expressed NifB proteins were similar or better than those reported for these variants when purified from Escherichia coli (18, 30). This result indicates that NifB*^Mi^*, NifB*^Ma^*, and NifB*^Mt^*, when expressed and isolated from tobacco organelles, are fully functional in NifB-co synthesis.

## DISCUSSION

The minimal gene set that must be transferred to plants to generate potentially active nitrogenase consists of *nifH*, *nifD*, *nifK*, *nifB*, *nifE*, and *nifN*, where *nifH*, *nifD*, and *nifK* encode the nitrogenase components and *nifB*, *nifE*, and *nifN* encode proteins essential for FeMo-co synthesis (24). Of these six genes, NifB is the only protein essential to all nitrogenases because it produces a metallocluster (NifB-co) representing the first committed step in the biosynthesis of FeMo-co, FeV-co, and FeFe-co, the active-site cofactors of all nitrogenases. Thus, NifB is a primary bottleneck to engineer a plant nitrogenase.

As for most proteins, solubility is a prerequisite for NifB activity. Previous studies had shown that NifB accumulated mostly as insoluble protein in heterologous hosts, even in bacteria (18, 30, 43). For this reason, together with the essential nature of the reaction performed by NifB, its engineering in plants has been a major bottleneck for the generation of nitrogen fixing crops. However, encouraging work in yeast showed that the *M. infernus* NifB was much more soluble in mitochondria than the A. vinelandii NifB (28). This indicated that solubility was not uniform for all NifB variants and prompted us to screen NifB expression libraries in mitochondria and chloroplasts, the two plant organelles proposed to host an engineered plant nitrogenase. Most tobacco expressed NifB proteins showed poor solubility in both chloroplasts and mitochondria. Only the three archaeal NifB variants that were included in the library accumulated as soluble proteins at high levels either in mitochondria or in chloroplasts.

The thermophilic nature of *M. infernus* and *M. thermautotrophicus* could explain why their NifB proteins were soluble and stable at the temperatures that can arise in mitochondria (44). In a similar study, a NifH variant from the thermophile Hydrogenobacter thermophilus excelled over other NifH proteins in solubility and accumulation when expressed in tobacco mitochondria (32). We are not aware of studies reporting temperature inside chloroplasts, but there are reports of *in vitro* Rubisco activity increasing 6-fold when raising assay temperature from 25 to 50°C, although a reduction of its activity *in vivo* has been observed above 37°C (45). The fact that Rubisco is catalytically active at such temperatures suggests that chloroplasts can also reach temperatures well above the mesophilic range of most diazotrophs, which could explain why the thermophilic NifB proteins remained soluble and were stable also in this organelle.

The yields of NifB*^Mi^* purifications were similar for tobacco chloroplasts and mitochondria. This was unexpected since chloroplasts are generally regarded superior for accumulating larger amounts of heterologous proteins. One study reported recombinant protein expression from modified chloroplast genome reaching 40 to 50% of total soluble protein without severely hampering photosynthetic efficiency (46). Similarly, nuclear expression of GFP has shown that chloroplast targeting (the strategy followed in our work) increased the yield to approximately 10% of total soluble protein compared to 0.5% when the GFP was maintained in the cytoplasm and nucleoplasm (47).

Although different experimental procedures make direct comparisons difficult, our results suggest that NifB activities are similar in tobacco and bacteria. For example, E. coli purified His-tagged NifB*^Mt^* and NifB*^Ma^* generated ARA activities of 250 (NifB*^Mt^*) and 750 (NifB*^Ma^*) units after *in vitro* chemical reconstitution of the NifB [Fe_4_S_4_] clusters (30), and NifB*^Mi^* produced in a similar way rendered ARA activities of 180 units (18). When these NifB variants were isolated from mitochondria and chloroplasts of tobacco leaves, they were all active *in vitro* when provided with [Fe_4_S_4_] clusters by NifU, and in all three cases the activities we observed were almost identical to those reported for the E. coli expressed proteins. The significantly higher activity obtained using NifB*^Ma^* could be explained by the FeMo-co synthesis assay being performed at 30°C, which is much closer to the optimal growth temperature reported for *M. acetivorans* (37°C) (48) compared to that of the thermophilic organisms *M. infernus* (85°C) and *M. thermautotrophicus* (65°C) (49, 50). Mesophilic NifB*^Ma^* could therefore be more efficient in NifB-co formation and transfer at 30°C than the thermophilic NifB*^Mt^* and NifB*^Mi^* proteins. However, whether the same difference in activity would be observed *in vivo*, considering the temperatures inside mitochondria and chloroplasts as discussed above, remains to be tested.

Although expression of the NifB proteins in A. vinelandii was difficult to validate (absence or weak signal when probing for the TS-tag), *in vivo* complementation by some NifB variants confirmed that expression was sufficient to support nitrogenase activity. Another limitation of our study is that the codon usage of the *nifB* genes was not optimized for A. vinelandii. Nevertheless, the number of NifB variants that could revert the Nif^–^ phenotype in the *in vivo* Δ*nifB* complementation is encouraging for future efforts to create N_2_-fixing plants. The lack of sequence similarity between the A. vinelandii NifB protein and that of the complementing NifB variants suggested that, if NifB is functional and produces NifB-co, the cluster is transferred to NifX/NifEN without the need of constructive protein interactions. It also indicates that the A. vinelandii NifU scaffold protein serves [Fe_4_S_4_] cluster substrates to a great variety of NifB acceptor proteins. This result shows that the maturation of nitrogenase in a heterologous host can be divided in independent modules. Thus, rather than identifying a unique source (organism) for the full Nif pathway, Nif proteins showing superior solubility, stability and functionality can be selected from very distinct origins.

In conclusion, the natural genetic diversity of diazotrophs can be exploited to overcome solubility and stability issues associated with Nif protein expression in eukaryotic cells. A screening-based experimental design serves to identify adequate Nif protein components for the engineering of a eukaryotic nitrogenase. In this work, functional NifB proteins have been obtained from tobacco mitochondria (*M. thermautotrophicus* and *M. infernus*) and chloroplasts (*M. acetivorans* and *M. infernus*). NifB yields were similar for both organelles. Although the as-isolated proteins did not exhibit color typical of NifB with full complement of [Fe_4_S_4_] clusters, all as-isolated NifB proteins were active *in vitro* when reconstituted with [Fe_4_S_4_] clusters by their natural cluster donor NifU. This either indicates that cluster-loading *in vivo* is poor or that the activity of NifB in mitochondria and chloroplasts is high (depleting cluster substrates and causing NifB to exist mainly in the form containing only the catalytic RS cluster). Due to the small amount of protein isolated from tobacco, we could not perform electron paramagnetic resonance to determine the Fe-S cluster composition of as-isolated NifB. As the stability of the NifB-co product is low, further studies using NifB-co acceptor proteins (e.g., NifX or NifEN) are needed to distinguish between these two possibilities. We have previously shown that NifH expressed in tobacco mitochondria was mainly isolated devoid of its [Fe_4_S_4_] cluster (32), suggesting that cluster biosynthesis, transfer or stability might be affected, and that future research in this area will be necessary. However, it is important to note that selected NifB proteins supported *in vitro* FeMo-co synthesis using [Fe_4_S_4_] cluster substrates provided by the physiological donor NifU, proving that these identified variants were isolated as functional proteins from both plant organelles.

Irrespectively of the type of nitrogenase being engineered in a N_2_-fixing plant (Mo, V, or Fe-only nitrogenase), the expression of functional NifB is crucial as it produces the NifB-co required for the synthesis of the active-site clusters FeMo-co, FeV-co, or FeFe-co. The identification of soluble and functional NifB variants expressed *in planta* is therefore crucial to the engineering of the nitrogenase cofactor biosynthesis, and hence the expression of active nitrogenase in plants.

## MATERIALS AND METHODS

### Phylogenetic trees.

Taxonomic IDs from selected organisms for the NifB library (see Table S2) were retrieved from the NCBI Taxonomy browser (51), and the resulting compilation data were exported to display the phylogenetic tree of selected organisms using iTOL (52). The MAFFT online service was used to align the NifB protein sequences (53), and the resulting alignment file was analyzed using IQ-TREE (54). ModelFinder (55) and Ultrafast Bootstrap approximation (56) were used to construct the maximum likelihood tree. Results were plotted using iTOL.

### Strains, media, and growth conditions.

E. coli DH5α was used for cloning of plasmid libraries (see section below) and was grown in lysogenic broth (LB) at 37°C with appropriate antibiotics. A. tumefaciens GV3101 (pMP90) used for agroinfiltration experiments was grown in LB supplemented with rifampicin (25 μg/mL) and gentamicin (10 μg/mL) plus appropriate antibiotics for maintaining the binary plasmids at 28°C. A. vinelandii strains were cultivated in Burk’s modified medium (containing 28 mM ammonium acetate) or in Burk’s modified N-free medium at 30°C (57) supplemented with appropriate antibiotics at standard concentrations (58).

A. tumefaciens used for tobacco infiltration experiments were freshly transformed and selected on solid LB medium with appropriate antibiotics at 28°C. Three days prior to leaf infiltration, a preinoculum was started from freshly transformed cells and incubated overnight at 28°C with shaking at 150 rpm. The inoculum was refreshed 1 day before infiltration at an optical density at 600 nm (OD_600_) of 0.01. At the day of infiltration, A. tumefaciens cultures (at an OD_600_ of ≥3.0) were transferred to induction medium containing 10 mM Mg_2_SO_4_, 10 mM 2-(N-morpholino)ethanesulfonic acid (MES) and 150 μM acetosyringone. Cells were induced at a final OD_600_ of 0.9 for 2 h and then used for infiltration into tobacco leaves using a syringe.

### *A. vinelandii* UW140 complementation.

The A. vinelandii strains DJ (wild type) and UW140 (Δ*nifB*) have been described (15). The NifB plasmid library (containing *nifB* genes with codon usage optimized for expression in Saccharomyces cerevisiae) generated in this study for complementing UW140 strain are listed in Table S1. A. vinelandii strains were grown in Burk medium at 30°C with shaking at 200 rpm. The procedure for A. vinelandii transformation has been described (59). Transformed cells were cultured overnight and then plated on nitrogen-free Burk solid medium supplemented with ampicillin (25 μg/mL) to select cells with plasmid integrated in the chromosome.

For *in vivo* ARA, A. vinelandii strains were grown in liquid Burk medium overnight, washed with nitrogen-free Burk medium, and then used to establish fresh cultures at an initial OD_600_ of 0.3 in baffled flasks. One milliliter of each culture was transferred to 9-mL serum vials after 4, 18, and 48 h of incubation. Vials were sealed with butyl rubber stoppers, and 500 μL of acetylene was injected. After 15 min at 30°C, the reactions were stopped by injecting 100 μL of 8 M NaOH. Ethylene formed was measured in 50-μL gas-phase samples using a Porapak N 80/100 column in a GC-2014 gas chromatograph system (Shimadzu).

### Tobacco growth and transient expression of transgenes.

N. benthamiana plants were grown in a greenhouse under 16:8 h (light:dark) cycle and watered weekly with 1 g/L Sequestrene G100 (Syngenta). Young leaves of 4- to 5-week-old tobacco plants were infiltrated using a 1-mL syringe containing A. tumefaciens cells in induction medium. At the end of the dark phase 4 days after infiltration, leaves were sampled for immunoblot analysis or purification experiments.

### Preparation of cell-free protein extracts for NifB solubility tests and protein purification.

Five-millimeter-diameter disks were sampled from infiltrated tobacco leaves, transferred to 2-mL Eppendorf tubes containing a steel ball 7 mm in diameter, and frozen in liquid N_2_. Leaf tissue was ground into fine powder using a lab vibration mill mixer (Qiagen Retsch MM300 TissueLyser). Total protein extracts were prepared by adding 2× Laemmli buffer to the leaf powder at a ratio of 2:1 (vol/wt) and heating for 10 min at 95°C. For soluble protein extracts, prechilled tobacco protein extraction buffer (28) was immediately added to each tube at a ratio of 2:1 (vol/wt) and then incubated for 30 min on spinning wheel at 4°C. Tubes were centrifuged at 14,000 rpm for 30 min at 4°C, and the resulting supernatants containing soluble proteins were transferred to new tubes containing 2× Laemmli buffer at a ratio of 1:1 (vol/vol) and then heated for 10 min at 80°C. Heat-denatured samples were centrifuged for 3 min at maximum speed in a table-top centrifuge prior to analysis by SDS-PAGE and immunoblotting.

NifB protein purifications were performed in anaerobic chambers (Coy System and MBraun) at <1 ppm O_2_. Typically, 100 g of agroinfiltrated tobacco leaves were snap-frozen in liquid N_2_ as previously described (32, 37). Frozen leaf material was transferred into the anaerobic chamber, and the tissue was homogenized using a blender (Classic 4655; Oster) with 100 mL of lysis buffer (100 mM Tris-HCl [pH 8.6], 200 mM NaCl, 10% glycerol, 5 mM β-mercaptoethanol, 2 mM sodium dithionite [DTH], 1 mM phenylmethylsulfonyl fluoride, 1 μg/mL leupeptin, 5 μg/mL DNase I). Homogenization was performed at maximum speed and maintained for 4 to 5 min. The resulting total cell extract was filtered through a cheese cloth to remove larger cell debris. The filtered cell extract was centrifuged using polypropylene tubes with a sealing cap at 53,250 × *g* in a JA 25.50 rotor (Beckman Coulter) for 1 h at 4°C. The supernatant was filtered through a 0.2-μm vacuum filter unit (Rapid-Flow; Nalgene) to obtain the cell extract (CFE) that was loaded into a Macroprep Strep-Tactin (IBA lifesciences) affinity column by gravity. After loading the extract, the column was washed with 10 column volumes of wash buffer (100 mM Tris-HCl [pH 8.0], 200 mM NaCl, 10% glycerol, 5 mM β-mercaptoethanol, 2 mM DTH). Bound protein was eluted from the column by applying 3 column volumes of wash buffer supplemented with 5 mM desthiobiotin (IBA Life Sciences), concentrated using Amicon centrifugal filter units with a 10-kDa cutoff (Merck Millipore), and then snap-frozen and stored in liquid N_2_.

### Protein techniques.

Antibodies used in this study and their dilutions for immunoblotting were as follows: antibodies detecting NifB, NifU and NifS were raised against A. vinelandii purified protein preparation in rabbit. Antibodies for the detection of HA tag (HA-HRP, 3F10, 12013819001; Roche), GFP (B-2, sc-9996; Santa Cruz), and TS-tag (Streptactin-HRP, 2-1502-001 [IBA Life Sciences] and StrepMAB, 2-1507-001 [IBA Life Sciences]) are commercially available. α-NifB, α-NifU, α-NifS, StrepMAB, and α-GFP were used at 1:3,000, 1:2,500, 1:2,500, 1:5,000 and 1:2,000 dilutions, respectively, in TBS-T supplemented with 5% bovine serum albumin (BSA) and 0.01% sodium azide. α-HA-HRP and Streptactin-HRP were used at 1:3,000 and 1:50,000 dilutions, respectively, in 2% skimmed milk in TBS-T. The secondary antibodies used for the detection of nonconjugated primary antibodies were α-rabbit-HRP (for α-NifB, α-NifU, and α-NifS) and α-mouse-HRP (for StrepMAB and α-GFP) at a 1:20,000 dilution.

For peptide mass fingerprinting analysis, Coomassie blue-stained protein bands were sliced with a scalpel blade and transferred to an Eppendorf tube with water. The service was provided by the Proteomic Unit from Universidad Complutense de Madrid.

For NifB N-terminal determination, samples were prepared according to the instruction provided by the service (Margarita Salas Center for Biological Research [CIBMS], Madrid, Spain). In short, 200 pmol of each protein variant was separated by SDS-PAGE, transferred to polyvinylidene difluoride membrane (Bio-Rad), and stained with Coomassie blue R-250. The bands corresponding to NifB were excised with a clean scalpel, dried, and transferred to an Eppendorf tube for analysis.

### *In vitro* reconstitution of *E. coli* NifU*^Av^*.

Plasmids encoding strep-tagged A. vinelandii NifU (pDB2174) and strep-tagged A. vinelandii NifS (pDB2123) were kindly donated by Dennis Dean (Virginia Polytechnic Institute and State University). The NifU purified from E. coli (designated NifU*^Ec^*) was reconstituted *in vitro* following the protocol as previously described (32, 60) with slight modifications. Next, a 20 μM concentration of NifU dimer was prepared in 100 mM morpholinepropanesulfonic acid (MOPS) buffer (pH 7.5) containing 8 mM 1,4-dithiothreitol (DTT) and incubated for 30 min at 37°C. To this mix, 1 mM l-cysteine, 1 mM DTT, 225 nM NifS dimer, and 0.3 mM (NH_4_)_2_Fe(SO_4_)_2_ were added. The iron was added in three steps with 15-min separations until reaching a final concentration of 0.3 mM. The reconstitution mix was kept on ice for 3 h and then desalted using centrifugal units with a 10-kDa cutoff (Amicon, Millipore) to replace the reaction mixture with 100 mM MOPS (pH 7.5). The [Fe_4_S_4_] cluster reconstituted NifU (called holo-NifU) was stored in liquid N_2_ until used.

### *In vitro* NifB-dependent FeMo-co synthesis and insertion assays using pure Nif proteins.

The NifB-dependent FeMo-co synthesis assays were performed under anaerobic conditions in 100-μL reaction mixtures containing 3.0 μM NifH*^Av^*, 1.0 μM tobacco purified NifB*^Xx^*, 1.5 μM apo-NifEN*^Av^*, 0.6 μM apo-NifDK*^Av^*, 17.5 μM Na_2_MoO_4_, 175 μM *R*-homocitrate, 9 μM holo-NifU*^Ec^*, 125 μM SAM, 1 mg/mL BSA, and ATP-regenerating mixture (1.23 mM ATP, 18 mM phosphocreatine disodium salt, 2.2 mM MgCl_2_, 3 mM DTH, 46 μg/mL creatine phosphokinase, final concentrations) in 100 mM MOPS buffer (pH 7.5) for 1 h at 30°C. NifB-co-dependent FeMo-co synthesis assays were performed as positive controls for NifB activity. These assays were performed as described above but without holo-NifU*^Ec^* and by replacing 1.0 μM NifB protein with 2.5 μM NifB-co.

The activity of reconstituted NifDK was determined after FeMo-co synthesis and insertion by adding 500 μL of ATP-regenerating mixture and 2.0 μM NifH*^Av^* and transferring the mixture to 9-mL rubber-stopped serum vials containing an Ar atmosphere and 500 μL of acetylene. The ARA was performed for 20 min at 30°C. After stopping the reactions by addition of 100 μL of 8 M NaOH, 50 μL of the gas phase was removed and injected into a GC-2014 gas chromatograph (Shimadzu) equipped with a Porapak N 80/100 column to measure ethylene formation.

### Data availability.

Accession numbers of genes used here are listed in Table S2.

10.1128/mbio.00268-22.5 TABLE S4 List of modular pieces and primers used for the generation of transcriptional units to assemble multigenic vectors using MoClo cloning. Download Table S4, PDF file, 0.1 MB. Copyright © 2022 Jiang et al. 2022 Jiang et al. https://creativecommons.org/licenses/by/4.0/ This content is distributed under the terms of the Creative Commons Attribution 4.0 International license.
