# Supplementary material for: Functional Nitrogenase Cofactor Maturase NifB in Mitochondria and Chloroplasts of Nicotiana benthamiana
Source: mBio. 2022 Jun 13;13(3):e00268-22. doi: 10.1128/mbio.00268-22 (PMC9239050; doi:10.1128/mbio.00268-22)
Supplement: TEXT S1 [file mbio.00268-22-s0001.pdf]

Materials and Methods

Design, cloning, and assembly of the *nifB* library

To generate the binary plasmid library for NifB expression and targeting to tobacco mitochondria, the parental vector pN2SB41 (containing a *pE35S::cox4-twinstrep-BamHI-gus-BstEII-tNOS*) (1) was digested with *BamHI* and *BstEII* to remove *gus* and insert the *nifB* genes (2) (Table S3). To generate the library containing NifB variants targeted to the chloroplast, a *pE35S::ssu-twinstrep-BamHI* fragment flanked by *HindIII* and *BglII* was synthesized by ThermoFisher via the Engineering Nitrogen Symbiosis for Africa (ENSA) project. The pGFPGUSplus plasmid (Addgene #64401) and the *pE35S::ssu-twinstrep-BamHI* DNA fragment were digested with *HindIII* and *BglII* and ligated to generate plasmid pN2SB42. The resulting *pE35S::ssu-twinstrep-BamHI-gus-BstEII-tNOS* construct of pN2SB42 mimicked the genetic structure of the mitochondria targeting parental plasmid pN2SB41. Previously *BamHI* and *BstEII* digested *nifB genes* were inserted into pN2SB42 digested with the same restriction enzymes, generating the plasmid library for chloroplast NifB targeting (Table S3).

The pGFPGUSplus was also used as parental plasmid to generate vectors for the expression of chloroplasts or mitochondria targeted NifB accessory proteins. To generate pN2XJ163, containing mitochondria targeted NifU, NifS and FdxN, the *su9-nifU* and *su9-nifS* genes were amplified by PCR from the vector pN2GLT4 (3) using primers AAAAGGATCCAATGGCCTCCACTCGTGTCCTCG and AAAAAAGGTCACCTTAGACTTCCATTTGGGCGTGTGCG inserting the flanking restriction sites for *BamHI* and *BstEII* (for *su9-nifU*), and AAACTAGTATGGCCTCCACTCGTGTCCTCG and AAAAGAGCTCTTAACCATAGACAGGAGCAAAGGCTTTACC inserting the flanking restriction sites for *SpeI* and *SacI* (for *su9-nifS*). The *su9-fdxN-HA* gene fragment was amplified by PCR from the vector pN2SB39 (4) using primers CTCTACAAATCTATCTCTCTCGAGATGGCCTCCACTCGTG and TATTATGGAGAAACTCGAGTTAAGCATAATCTGGAACATC inserting *XhoI* sites. pGFPGUSplus was digested with *BglII* and *BstEII* to insert *su9-nifU*, then digested with *XbaI* and *SacI* to insert *su9-nifS* using Ligase T4. Finally, *su9-fdxN-HA* was inserted by digestion of the plasmid with *XhoI* and inserted by exonuclease and ligation independent cloning (5).

To generate pN2XJ164 containing chloroplasts targeted NifU, NifS and FdxN, an overlapping PCR approach was used to merge the sequence for *ssu* to *nifU, nifS and fdxN-HA*. Primer combinations AAAAAAGGATCCAATGGCTTCCTCAGTTCTTTCC and CCTTTTCAGAGTAGTCCCACACCTGCATGCATTGCACTC (for *ssu*), with AAAAAAGGTCACCTTAGACTTCCATTTGGGCGTGTGCG and CAATGCATGCAGGTGTGGGACTACTCTGAAAAGGTTAAGG (for *nifU*), were used to create *ssu-nifU*. Primer combinations AAAAAAACTAGTATGGCTTCCTCAGTTCTTTCC and CAAGTAAACGTCGGCCACCTGCATGCATTGCACTCTTC (for *ssu*), with CTCTACAAATCTATCTCTCTCGAGATGGCTTCCTCAGTTC and ACTATCTTAAGAGCCATCACCTGCATGCATTGCACTCTTC (for *nifS*) were used to create *ssu-nifS*. Primer combinations GCAATGCATGCAGGTGGCCGACGTTTACTTGGATAATAAC and AAAAGAGCTCTTAACCATAGACAGGAGCAAAGGCTTTACC (for *ssu*), with TGCAATGCATGCAGGTGATGGCTCTTAAGATAGTTGAGTC and TATTATGGAGAAACTCGAGTTAAGCATAATCTGGAACATC (for *fdxN-HA*) were used to create *ssu-fdxN-HA*. The *nifU*, *nifS* and *fdxN-HA* sequences were amplified using pN2XJ163 as template, and the *ssu* was amplified using pN2SB42 as template. The insertion sites for *ssu-nifU, ssu-nifS* and *ssu-fdxN-HA* used to generate plasmid pN2XJ164 were the same as for pN2XJ163.

The plasmid to integrate *nifB* genes into the *A. vinelandii* chromosome was a derivative of pRHB272 (6). pRHB272 contains homologous sequences for recombination downstream Avin02530, without disrupting any known functional gene sequence. A DNA fragment encoding the *ts*-tag followed by *BamHI* and *BstEII* restriction sites was introduced downstream of the *nifH* promoter in pRHB272 using *NdeI* and *EcoRI* restriction sites, generating the parental plasmid pN2SB51. pN2SB51 and the different *nifB* genes were digested with *BamHI* and *BstEII*. The *nifB* fragments were cloned into pN2SB51 using T4 Ligase (Promega), generating plasmids pN2XJ113-pN2XJ142 (Table S1) used to transform *A. vinelandii* UW140 strain (Δ*nifB*).

Correct gene insertion into plasmids and integrity of all inserted genes was confirmed by Sanger sequencing (Macrogen).

Multigenic vectors assembled using MoClo

Multigenic constructs were created using *nifB* sequences from *M. infernus* (plasmid pN2XJ21), *M. thermautotrophicus* (plasmid pN2XJ64) or *M. acetivorans* (plasmid pN2XJ63), together with the *fdxN-HA* (plasmid pN2X163), *nifU* and *nifS* genes from *A. vinelandii* (7) and the silencing suppressor *p19* (GB1203) (8) and *eGFP.* Vectors were designed in Geneious (9) and all parts were assembled using the Type IIS MoClo cloning system (10, 11). Standardized Level 0 phytobricks were produced as previously described (7) using primers and templates provided in Tables S3 and S4.

MoClo restriction-ligations were performed at 40:20 fmol ratio of each insert:acceptor vector in 20 µl reactions containing 5U of the required restriction enzyme (*BpiI*, Thermo Scientific) for Level 0 phytobricks and Level 2 constructs, and *BsaI*-HFv2 (NEB) for Level 1 constructs, together with 4.5U of T4 Ligase (Promega), 1.5 µl of 10x Ligase Buffer and 1.5 µl of 10x BSA (Canvax Biotech). The reactions were incubated in a thermocycler using the following program: 20 seconds at 37ºC, [3 minutes at 37ºC, 4 minutes at 16ºC] for 26 cycles, 5 minutes at 37ºC and finally 5 minutes at 80ºC.

Chemically competent *E. coli* TOP10 cells were transformed with reaction mixtures and then plated on selective LB solid medium containing 20 µg/ml X-Gal (Duchefa Biochemie) and 1 mM IPTG (Sigma-Aldrich), supplemented with 50 µg/ml spectinomycin (Sigma-Aldrich) for Level 0 phytobricks, 100 µg/ml carbenicillin (Formedium) for Level 1 constructs or kanamycin (Formedium) for Level 2 constructs. White colonies were selected for plasmid DNA extraction using the GenElute Plasmid Miniprep Kit (Sigma-Aldrich). The integrity of all plasmids was verified by Sanger sequencing (Macrogen, Eurofins Genomics). A list of all Level 1 and Level 2 constructs generated and used in this work can be found in Tables S3 and S4.

Vector targeting eGFP to tobacco chloroplasts

The *ssu* chloroplast targeting peptide encoding sequence was amplified by PCR from vector pN2SB42 using primers GGAGAGAACACGGGGGACTCTAGAATGGCTTCCTCAGTTC and CATGGATCGAATTGATCCTCTAGACACCTGCATGCATTGC, which insert *XbaI* restriction sites in the amplicon and introduce sequences homologous to those flanking *XbaI* sites in pGFPGUSplus. The pGFPGUSplus plasmid (Addgene #64401) was

digested with *XbaI* and the PCR amplified fragment was cloned into the vector by exonuclease and ligation independent cloning (5), generating pN2XJ158 (*p35S::ssu-eGFP-tnos*).

Confocal microscopy of *N. benthamiana* leaves

Subcellular localization of fluorescent protein tagged polypeptides SSU-eGFP and mito-RFP (12) was examined in *A. tumefaciens* infiltrated leaves of *N. benthamiana* using a Leica TCS SP8 laser scanning confocal microscope with a 40x/1.10 water immersion objective equipped with LAS X software (Leica). eGFP, RFP, and chlorophyll were excited with 488 nm, 561 nm, and 638 nm laser lines, respectively, with an emission band of 500 to 537 nm for eGFP detection, 585 to 620 nm for RFP detection, and 652 to 727 nm for chlorophyll autofluorescence. Chlorophyll and eGFP were recorded simultaneously, while RFP was detected in a separate scan. Laser intensity and gain was maintained during each experiment.

Figures

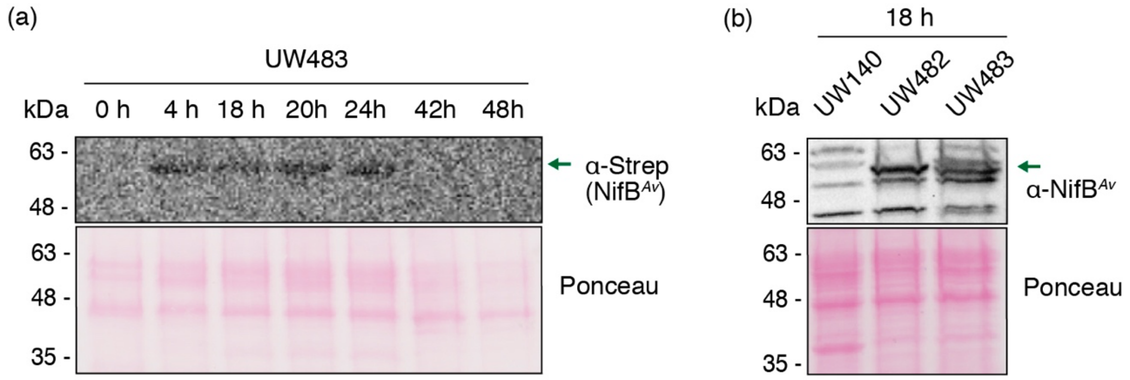

Figure S1. **Immunodetection of TS-NifB^{Av} expressed in *A. vinelandii* UW140 strain**. (a) Analysis of TS-NifB^{Av} expression in total protein extracts of *A. vinelandii* UW483 at different time-points after the start of derepression (0 h). Note that the exposure had to be enhanced digitally in order to detect signal. (b) Immunoblot analysis of total protein extracts from *A. vinelandii* UW140 (Δ*nifB*), UW482 (the wild-type strain DJ transformed with the parental empty vector P_{nifH}*::ts*) and UW483 (UW140 transformed with P_{nifH}*::ts-nifB*^{Av}) sampled 18 h after derepression using polyclonal NifB^{Av} antibodies.

(a)

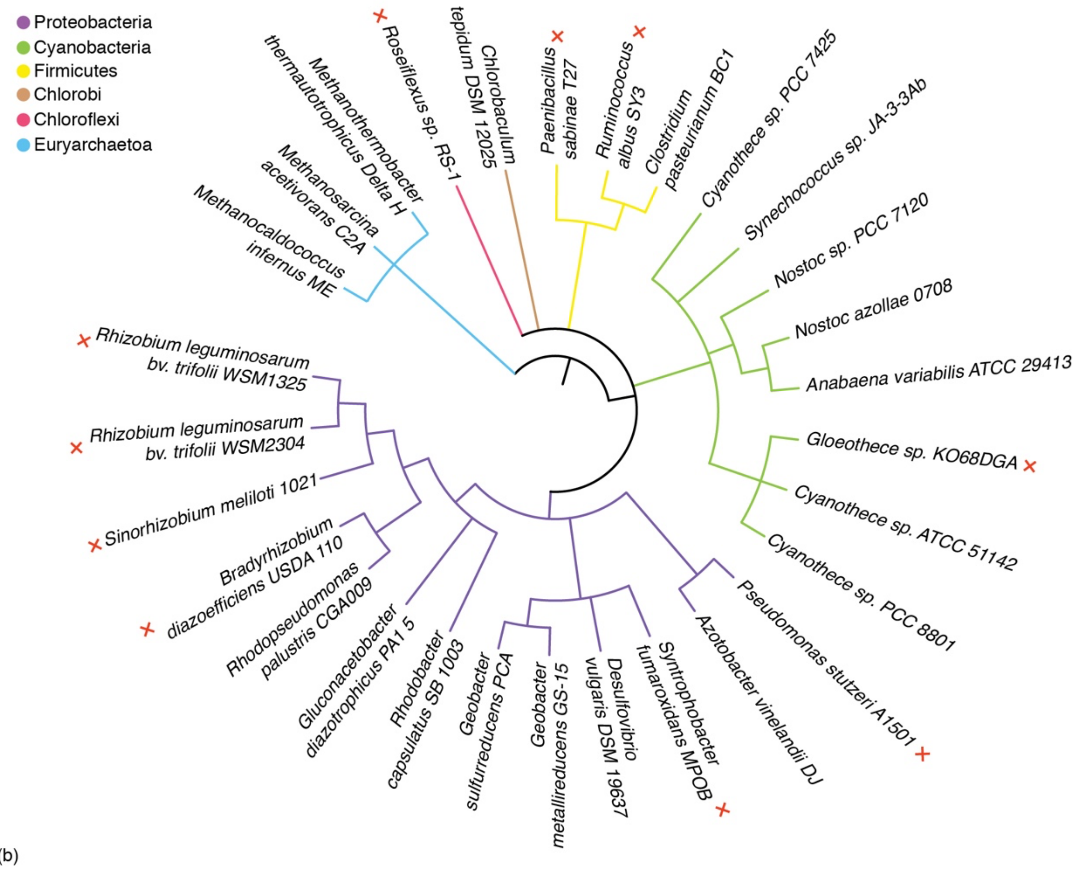
Proteobacteria
Cyanobacteria
Firmicutes
Chlorobi
Chloroflexi
Euryarchaetoa
Roseiflexus sp. RS-1
Chlorobaculum tepidum DSM 12025
Paenibacillus sabinae T27
Ruminococcus albus SY3
Clostridium pasteurianum BC1
Cyanothece sp. PCC 7425
Synechococcus sp. JA-3-3Ab
Nostoc sp. PCC 7120
Nostoc azollae 0708
Anabaena variabilis ATCC 29413
Gloeothece sp. KO68DGA
Cyanothece sp. ATCC 51142
Cyanothece sp. PCC 8801
Pseudomonas stutzeri A1501
Azotobacter vinelandii DJ
Syntrophobacter fumaroxidans MPOB
Desulfovibrio vulgaris DSM 19637
Geobacter metallireducens GS-15
Geobacter sulfurreducens PCA
Rhodobacter capsulatus SB 1003
Gluconacetobacter diazotrophicus PA1 5
Rhodopseudomonas palustris CGA009
Bradyrhizobium diazoefficiens USDA 110
Sinorhizobium meliloti 1021
Rhizobium leguminosarum bv. trifolii WSM2304
Rhizobium leguminosarum bv. trifolii WSM1325
Methanocaldococcus infernus ME
Methanosarcina acetivorans C2A
Methanothermobacter thermautotrophicus Delta H

(b)

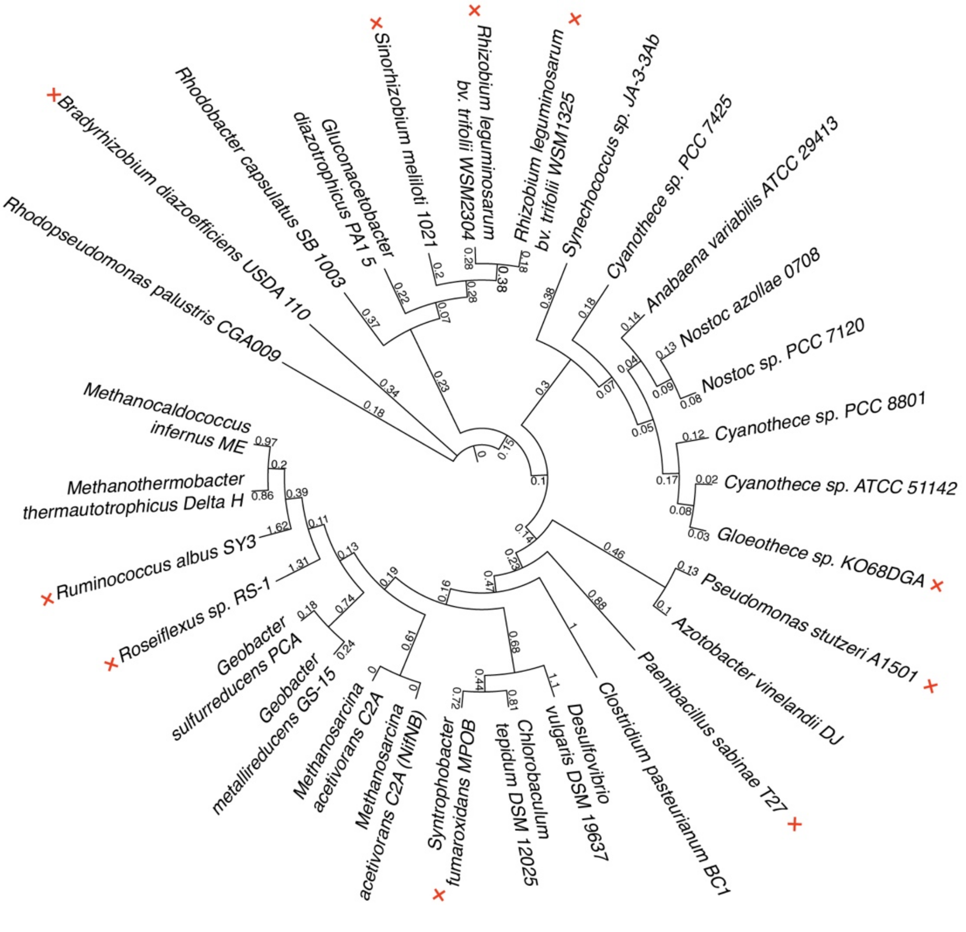
Sinorhizobium meliloti 1021
Rhizobium leguminosarum bv. trifolii WSM2304
Rhizobium leguminosarum bv. trifolii WSM1325
Synechococcus sp. JA-3-3Ab
Cyanothece sp. PCC 7425
Anabaena variabilis ATCC 29413
Nostoc azollae 0708
Nostoc sp. PCC 7120
Cyanothece sp. PCC 8801
Cyanothece sp. ATCC 51142
Gloeothece sp. KO68DGA
Pseudomonas stutzeri A1501
Azotobacter vinelandii DJ
Paenibacillus sabinae T27
Clostridium pasteurianum BC1
Desulfovibrio vulgaris DSM 19637
Chlorobaculum tepidum DSM 12025
Syntrophobacter fumaroxidans MPOB
Methanosarcina acetivorans C2A (NifNB)
Methanosarcina acetivorans C2A
Geobacter metallireducens GS-15
Geobacter sulfurreducens PCA
Roseiflexus sp. RS-1
Ruminococcus albus SY3
Methanothermobacter thermautotrophicus Delta H
Methanocaldococcus infernus ME
Rhodopseudomonas palustris CGA009
Bradyrhizobium diazoefficiens USDA 110
Rhodobacter capsulatus SB 1003
Gluconacetobacter diazotrophicus PA1 5

Figure S2. **Phylogenetic trees**. (a) Phylogenetic tree generated using NCBI Taxonomy Browser data of the species constituting the NifB library. Branch colors represent different phyla. (b) Maximum likelihood tree of the 30 NifB variants included in the library. Red crosses indicate variants unable to support growth of *ts*-*nifB* complemented *A*. *vinelandii* UW140 cells on nitrogen-free solid media.

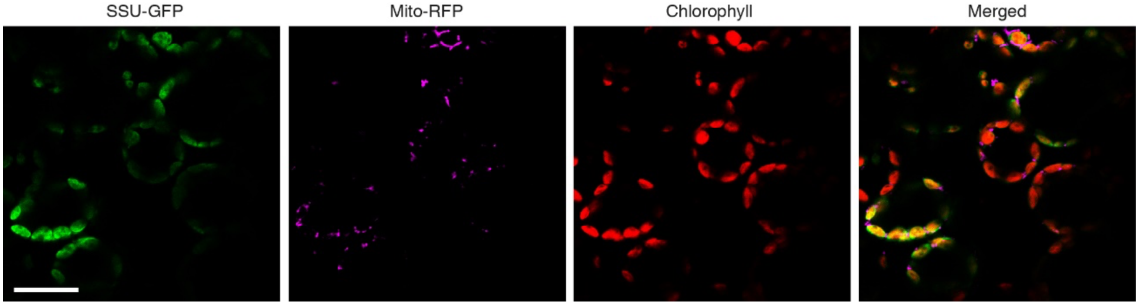

Figure S3. Chloroplast targeting of SSU-GFP in *N. benthamiana* leaf cells. Leaf cells co-expressing SSU-GFP (green) and the mitochondrial marker mito-RFP (magenta). Chlorophyll autofluorescence is shown in red. The merged image shows the overlap of GFP and chlorophyll in yellow. Scale bar size is 30 µm.

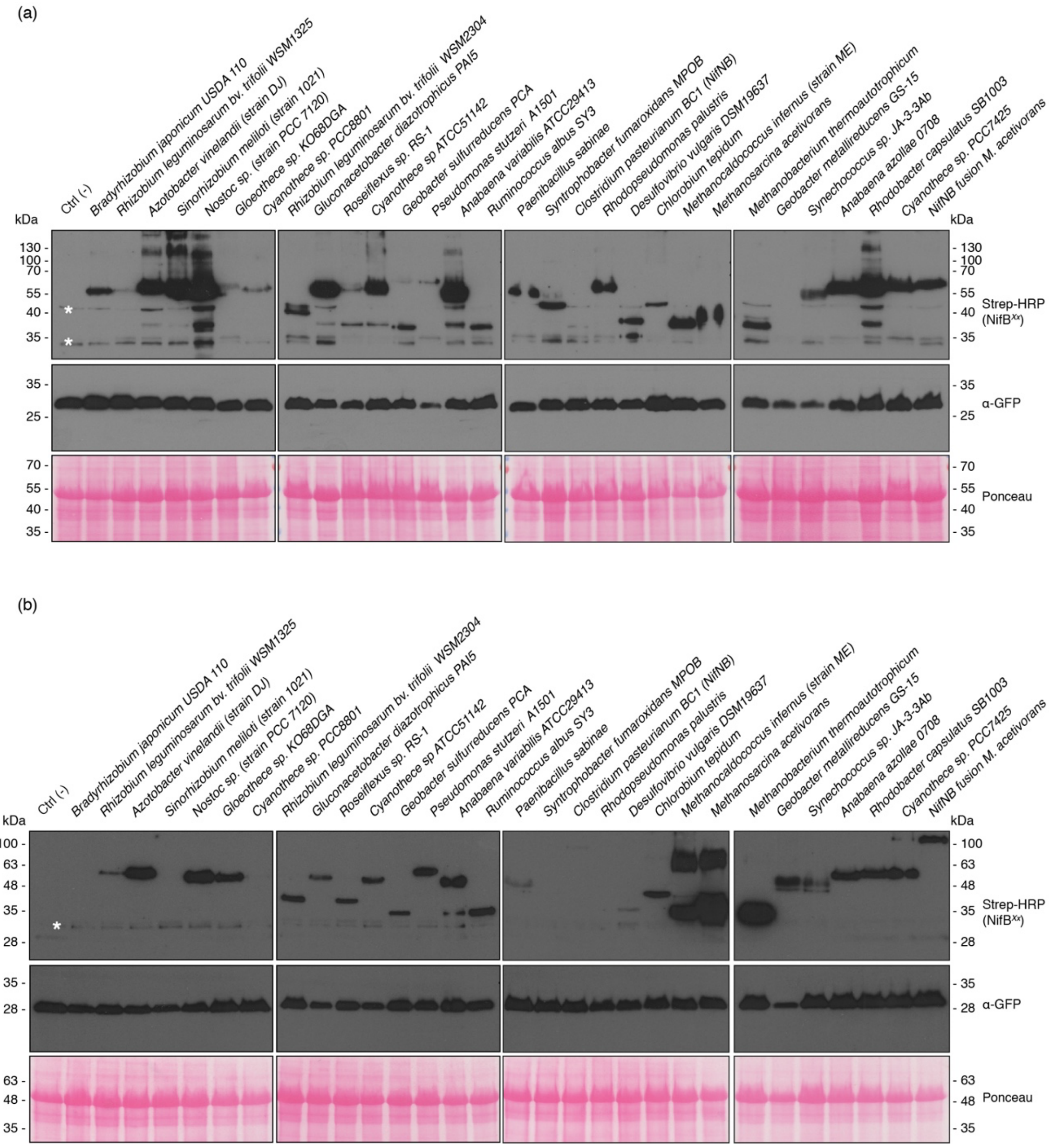

Figure S4. **Screening of NifB expression in mitochondria and chloroplasts of tobacco**. Immunoblot analysis using Streptactin-HRP to show accumulation of TS-NifB variants in total protein extracts of tobacco leaves. Upon transient expression, NifB variants were targeted to either mitochondria (a) or chloroplasts (b). GFP accumulation in the cytosol detected with anti-GFP antibodies is shown as infiltration control. *N. benthamiana* leaves were co-infiltrated with a mixture of *A. tumefaciens* strains containing plasmids for expression of NifU, NifS and FdxN-HA (pN2XJ163 and pN2XJ164) and p19 (GB1203), in addition to the NifB variants and GFP (Table S3). Ctrl (-) is a sample of tobacco infiltrated with pN2XJ163 or pN2XJ164 and pGFPGUSplus. The figure shows representative immunoblots of at least three independent infiltration experiments. White asterisks indicate unspecific bands.

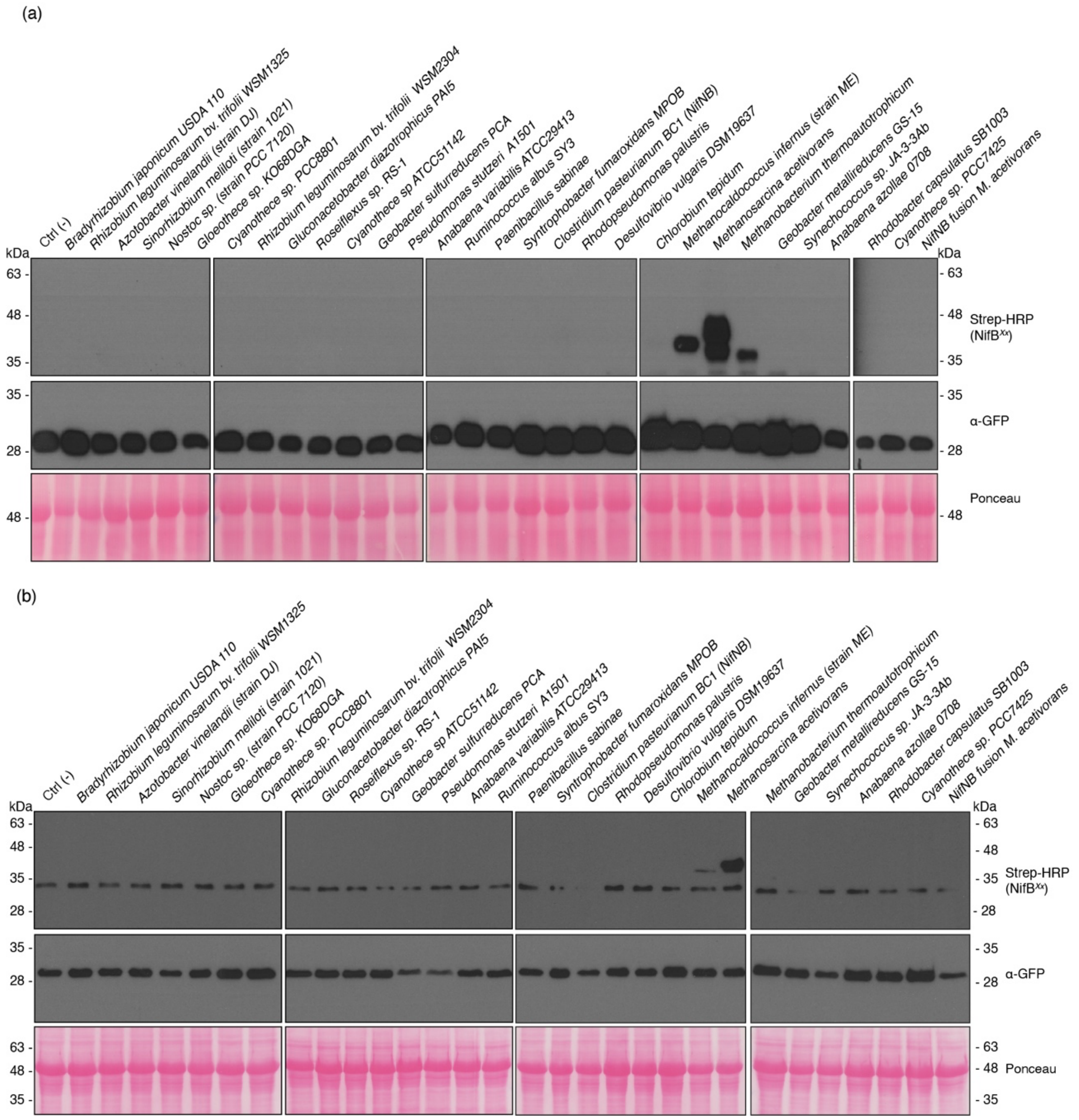

Figure S5. **Screening of NifB solubility in tobacco mitochondria and chloroplasts**. Immunoblot analysis using Streptactin-HRP to show accumulation of TS-NifB variants in soluble extracts of tobacco leaves. Upon transient expression, NifB variants were targeted to either mitochondria (a) or chloroplasts (b). GFP accumulation in the cytosol detected with anti-GFP antibodies is shown as infiltration control. *N. benthamiana* leaves were co-infiltrated with a mixture of *A. tumefaciens* strains containing plasmids for expression of NifU, NifS and FdxN-HA (pN2XJ163 and pN2XJ164 plasmids) and p19 (GB1203), in addition to the NifB variants and GFP (Table S3). Ctrl (-) is a sample of tobacco infiltrated with pN2XJ163 or pN2XJ164 and pGFPGUSplus. The figure shows representative immunoblots of three independent infiltration experiments.

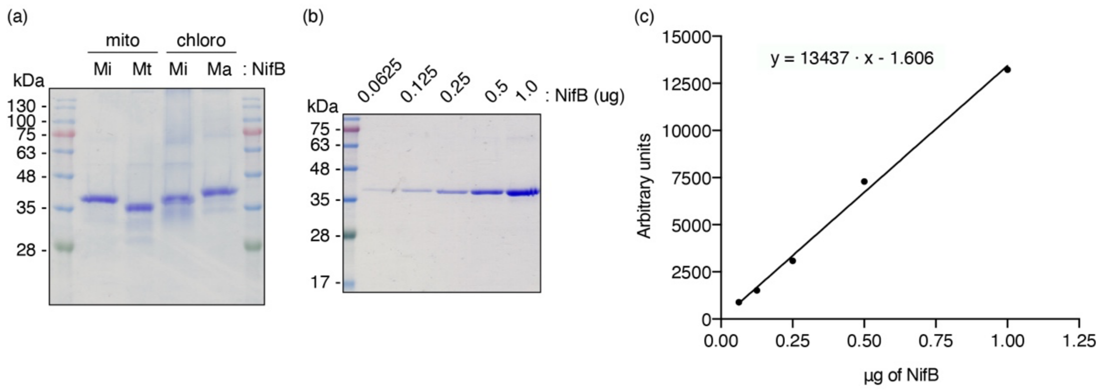

Figure S6. **Quantification of tobacco-purified NifB variants using yeast-purified NifB^{Mi} protein standard**. (a) Coomassie staining of purified NifB^{Xx} variants. A representative of each NifB variant per organelle is shown. (b) Coomassie staining of known and increasing amounts of NifB^{Mi} purified from yeast mitochondria. Both gels were loaded at the same time, run under the same electrophoresis parameters, and equally stained and destained. (c) Standard curve generated from NifB band intensity of gel in panel b, obtained using ImageJ ($R^2 = 0.995$). Generated equation was used to estimate the amounts of NifB proteins in the gel in panel a.

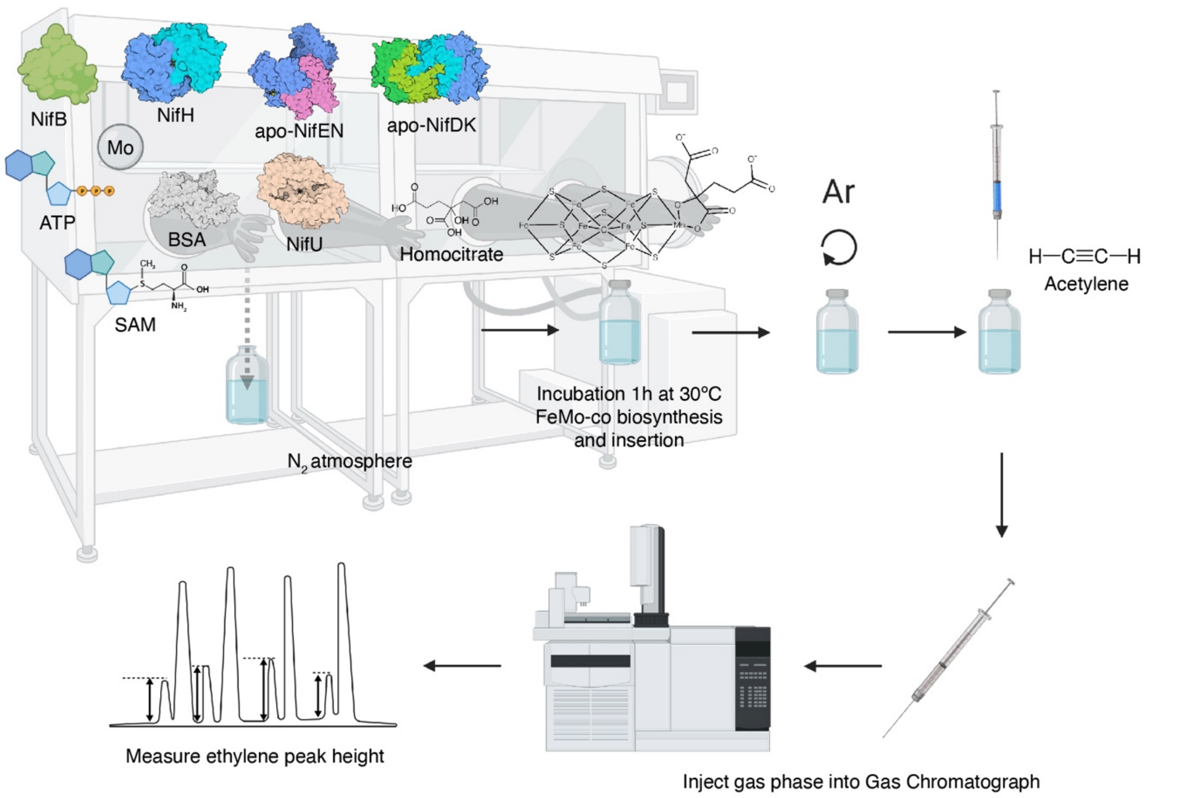

Figure S7. ***In vitro* FeMo-co synthesis and insertion assay procedure**. Representation of the steps followed to perform the NifB-dependent FeMo-co synthesis and insertion into apo-NifDK using purified Nif proteins from tobacco and *A. vinelandii*. Firstly, NifB and the substrates required for NifB-co formation (holo-NifU, SAM and reducing power) were mixed with other purified Nif proteins and substrates that mature NifB-co to FeMo-co (apo-NifEN, NifH, molybdate, *R*-homocitrate, and an ATP regenerating mix), and with apo-NifDK that receives *de novo* synthesized FeMo-co. Synthesis and insertion of FeMo-co into apo-NifDK was performed under anaerobic atmosphere. After that, the mix was transferred to vials, sealed, and the gas phase was exchanged to argon. Acetylene was injected into the argon gas phase, vials were incubated at 30ºC and reaction stopped before measuring ethylene formation in a gas chromatograph.

References

1. Jiang X, Paya-Tormo L, Coroian D, Garcia-Rubio I, Castellanos-Rueda R, Eseverri A, Lopez-Torrejon G, Buren S, Rubio LM. 2021. Exploiting genetic diversity and gene synthesis to identify superior nitrogenase NifH protein variants to engineer N_2-fixation in plants. Commun Biol 4:4.
2. Buren S, Pratt K, Jiang X, Guo Y, Jimenez-Vicente E, Echavarri-Erasun C, Dean DR, Saaem I, Gordon DB, Voigt CA, Rubio LM. 2019. Biosynthesis of the nitrogenase active-site cofactor precursor NifB-co in *Saccharomyces cerevisiae*. Proc Natl Acad Sci U S A 116:25078-25086.
3. Lopez-Torrejon G, Jimenez-Vicente E, Buesa JM, Hernandez JA, Verma HK, Rubio LM. 2016. Expression of a functional oxygen-labile nitrogenase component in the mitochondrial matrix of aerobically grown yeast. Nat Commun 7:11426.
4. Buren S, Jiang X, Lopez-Torrejon G, Echavarri-Erasun C, Rubio LM. 2017. Purification and *in vitro* acivity of mitochondria targeted nitrogenase cofactor maturase NifB. Front Plant Sci 8:1567.
5. Koskela EV, Frey AD. 2015. Homologous recombinatorial cloning without the creation of single-stranded ends: exonuclease and ligation-independent cloning (ELIC). Mol Biotechnol 57:233-40.
6. Hernandez JA, Curatti L, Aznar CP, Perova Z, Britt RD, Rubio LM. 2008. Metal trafficking for nitrogen fixation: NifQ donates molybdenum to NifEN/NifH for the biosynthesis of the nitrogenase FeMo-cofactor. Proc Natl Acad Sci U S A 105:11679-84.
7. Eseverri A, Lopez-Torrejon G, Jiang X, Buren S, Rubio LM, Caro E. 2020. Use of synthetic biology tools to optimize the production of active nitrogenase Fe protein in chloroplasts of tobacco leaf cells. Plant Biotechnol J 18:1882-1896.
8. Naim F, Nakasugi K, Crowhurst RN, Hilario E, Zwart AB, Hellens RP, Taylor JM, Waterhouse PM, Wood CC. 2012. Advanced engineering of lipid metabolism in *Nicotiana benthamiana* using a draft genome and the V2 viral silencing-suppressor protein. PLoS One 7:e52717.
9. Kearse M, Moir R, Wilson A, Stones-Havas S, Cheung M, Sturrock S, Buxton S, Cooper A, Markowitz S, Duran C, Thierer T, Ashton B, Meintjes P, Drummond A. 2012. Geneious Basic: an integrated and extendable desktop

software platform for the organization and analysis of sequence data. Bioinformatics 28:1647-9.

10. Weber E, Engler C, Gruetzner R, Werner S, Marillonnet S. 2011. A modular cloning system for standardized assembly of multigene constructs. PLoS One 6:e16765.

11. Werner S, Engler C, Weber E, Gruetzner R, Marillonnet S. 2012. Fast track assembly of multigene constructs using Golden Gate cloning and the MoClo system. Bioeng Bugs 3:38-43.

12. Candat A, Paszkiewicz G, Neveu M, Gautier R, Logan DC, Avelange-Macherel M-H, Macherel D. 2014. The Ubiquitous Distribution of Late Embryogenesis Abundant Proteins across Cell Compartments in Arabidopsis Offers Tailored Protection against Abiotic Stress. The Plant Cell 26:3148-3166.
