# Supplementary material for: Functional Nitrogenase Cofactor Maturase NifB in Mitochondria and Chloroplasts of Nicotiana benthamiana
Source: mBio. 2022 Jun 13;13(3):e00268-22. doi: 10.1128/mbio.00268-22 (PMC9239050; doi:10.1128/mbio.00268-22)
Supplement: TABLE S1 [file mbio.00268-22-s0003.pdf]

Table S1. List of vectors used to complement *A. vinelandii* UW140 (Δ*nifB*).

Strains	Plasmids	Origin of expressed TS-NifB protein	Complementation of Δ*nifB*
-	pN2XJ113	*Bradyrhizobium diazoefficiens USDA110*	-
-	pN2XJ114	*Rizhobium leguminosarum bv trifolii WSM1325*	-
UW483	pN2XJ115	*Azotobacter vinelandii DJ*	+
-	pN2XJ116	*Sinorhizobium meliloti 1021*	-
UW484	pN2XJ117	*Nostoc sp. PCC 7120*	+
-	pN2XJ118	*Gloeothece sp. KO68DGA*	-
UW485	pN2XJ119	*Cyanothece sp. PCC 8801*	+
-	pN2XJ120	*Rhizobium leguminosarum bv. trifolii WSM2304*	-
UW486	pN2XJ121	*Gluconacetobacter diazotrophicus PAl 5*	+
-	pN2XJ122	*Roseiflexus sp. RS-1*	-
UW500	pN2XJ123	*Cyanothece sp. ATCC 51142*	+
UW487	pN2XJ124	*Geobacter sulfurreducens PCA*	+
-	pN2XJ125	*Pseudomonas stutzeri A1501*	-
UW488	pN2XJ126	*Anabaena variabilis ATCC 29413*	+
-	pN2XJ127	*Ruminococcus albus SY3*	-
-	pN2XJ128	*Paenibacillus sabinae T27*	-
-	pN2XJ129	*Syntrophobacter fumaroxidans MPOB*	-
UW489	pN2XJ130	*Clostridium pasteurianum BC1*	+
UW490	pN2XJ131	*Rhodopseudomonas palustris CGA009*	+
UW491	pN2XJ132	*Desulfovibrio vulgaris DSM19637*	+
UW492	pN2XJ133	*Chlorobium tepidum DSM 12025*	+
UW493	pN2XJ134	*Methanocaldococcus infernus DSM11812*	+
UW494	pN2XJ135	*Methanosarcina acetivorans DSM 2834*	+
UW501	pN2XJ136	*Methanothermobacter thermautotrophicus DSM1053*	+
UW495	pN2XJ137	*Geobacter metallireducens GS-15*	+
UW496	pN2XJ138	*Synechococcus sp. JA-3-3A*	+
UW497	pN2XJ139	*Nostoc azollae strain 0708*	+
UW502	pN2XJ140	*Cyanothece sp. PCC 7425*	+
UW498	pN2XJ141	*Rhodobacter capsulatus SB 1003*	+
UW499	pN2XJ142	*Methanosarcina acetivorans DSM 2834 (NifNB fusion)*	+
