# Supplementary material for: Functional Nitrogenase Cofactor Maturase NifB in Mitochondria and Chloroplasts of Nicotiana benthamiana
Source: mBio. 2022 Jun 13;13(3):e00268-22. doi: 10.1128/mbio.00268-22 (PMC9239050; doi:10.1128/mbio.00268-22)
Supplement: TABLE S3 [file mbio.00268-22-s0004.pdf]

Table S3. Plant binary vectors used in this study.

Plasmids	Expressed proteins	Promoter	Size (kDa) FL	Size (kDa) P
pN2XJ163	SU9-NifU^{Av}	p35S	40.8	33.6
	SU9-NifS^{Av}	p35S	51.3	44.0
	SU9-FdxN^{Av}-HA	pE35S	18.2	10.7
pN2XJ164	SSU-NifU^{Av}	p35S	39.7	33.6
	SSU-NifS^{Av}	p35S	50.1	44.0
	SSU-FdxN^{Av}-HA	pE35S	16.8	10.7
GB1203	p19	p35S	19.4	-
pN2XJ20	COX4-TS-NifB$^{Azotobacter\ vinelandii\ DJ}$	pE35S	61.4	57.9
	eGFP	p35S	27.0	-
pN2XJ79	SSU-TS-NifB$^{Azotobacter\ vinelandii\ DJ}$	pE35S	64.0	57.9
	eGFP	p35S	27.0	-
pN2XJ21	COX4-TS-NifB$^{Methanocaldococcus\ infernus\ DSM11812}$	pE35S	41.7	38.2
	eGFP	p35S	27.0	-
pN2XJ80	SSU-TS-NifB$^{Methanocaldococcus\ infernus\ DSM11812}$	pE35S	44.3	38.2
	eGFP	p35S	27.0	-
pN2XJ22	COX4-TS-NifB$^{Bradyrhizobium\ diazoefficiens\ USDA110}$	pE35S	61.1	58.1
	eGFP	p35S	27.0	-
pN2XJ23	SSU-TS- NifB$^{Bradyrhizobium\ diazoefficiens\ USDA110}$	pE35S	64.2	58.1
	eGFP	p35S	27.0	-
pN2XJ24	COX4-TS-NifB$^{Rizhobium\ leguminosarum\ bv\ trifolii\ WSM1325}$	pE35S	60.3	56.8
	eGFP	p35S	27.0	-
pN2XJ25	SSU-TS-NifB$^{Rizhobium\ leguminosarum\ bv\ trifolii\ WSM1325}$	pE35S	62.9	56.8
	eGFP	p35S	27.0	-
pN2XJ26	COX4-TS-NifB$^{Sinorhizobium\ meliloti\ 1021}$	pE35S	60.9	57.4
	eGFP	p35S	27.0	-
pN2XJ27	SSU-TS-NifB$^{Sinorhizobium\ meliloti\ 1021}$	pE35S	63.5	57.4
	eGFP	p35S	27.0	-
pN2XJ28	COX4-TS-NifB$^{Nostoc\ sp.\ PCC\ 7120}$	pE35S	59.6	56.1
	eGFP	p35S	27.0	-
pN2XJ29	SSU-TS-NifB$^{Nostoc\ sp.\ PCC\ 7120}$	pE35S	62.2	56.1
	eGFP	p35S	27.0	-
pN2XJ30	COX4-TS-NifB$^{Gloeothece\ sp.\ KO68DGA}$	pE35S	60.8	57.3
	eGFP	p35S	27.0	-
pN2XJ31	SSU-TS-NifB$^{Gloeothece\ sp.\ KO68DGA}$	pE35S	63.4	57.3
	eGFP	p35S	27.0	-
pN2XJ32	COX4-TS-NifB$^{Cyanothece\ sp.\ PCC\ 8801}$	pE35S	60.7	57.2
	eGFP	p35S	27.0	-
pN2XJ33	SSU-TS-NifB$^{Cyanothece\ sp.\ PCC\ 8801}$	pE35S	63.3	57.2
	eGFP	p35S	27.0	-

pN2XJ34	COX4-TS-NifB$^{\textit{Rhizobium leguminosarum bv. trifolii WSM2304}}$	pE35S	46.7	43.2
	eGFP	p35S	27.0	-
pN2XJ35	SSU-TS-NifB$^{\textit{Rhizobium leguminosarum bv. trifolii WSM2304}}$	pE35S	49.3	43.2
	eGFP	p35S	27.0	-
pN2XJ36	COX4-TS-NifB$^{\textit{Gluconacetobacter diazotrophicus PAl 5}}$	pE35S	60.1	56.6
	eGFP	p35S	27.0	-
pN2XJ37	SSU-TS-NifB$^{\textit{Gluconacetobacter diazotrophicus PAl 5}}$	pE35S	62.7	56.6
	eGFP	p35S	27.0	-
pN2XJ38	COX4-TS-NifB$^{\textit{Roseiflexus sp. RS-1}}$	pE35S	41.1	37.6
	eGFP	p35S	27.0	-
pN2XJ39	SSU-TS-NifB$^{\textit{Roseiflexus sp. RS-1}}$	pE35S	43.7	37.6
	eGFP	p35S	27.0	-
pN2XJ40	COX4-TS-NifB$^{\textit{Cyanothece sp. ATCC 51142}}$	pE35S	60.9	57.4
	eGFP	p35S	27.0	-
pN2XJ41	COX4-TS- NifB$^{\textit{Cyanothece sp. ATCC 51142}}$	pE35S	63.5	57.4
	eGFP	p35S	27.0	-
pN2XJ42	COX4-TS-NifB$^{\textit{Geobacter sulfurreducens PCA}}$	pE35S	37.9	34.4
	eGFP	p35S	27.0	-
pN2XJ43	SSU-TS-NifB$^{\textit{Geobacter sulfurreducens PCA}}$	pE35S	40.5	34.4
	eGFP	p35S	27.0	-
pN2XJ44	COX4-TS-NifB$^{\textit{Pseudomonas stutzeri A1501}}$	pE35S	61.3	57.8
	eGFP	p35S	27.0	-
pN2XJ45	SSU-TS-NifB$^{\textit{Pseudomonas stutzeri A1501}}$	pE35S	63.9	57.8
	eGFP	p35S	27.0	-
pN2XJ46	COX4-TS-NifB$^{\textit{Anabaena variabilis ATCC 29413}}$	pE35S	60.7	57.2
	eGFP	p35S	27.0	-
pN2XJ47	SSU-TS-NifB$^{\textit{Anabaena variabilis ATCC 29413}}$	pE35S	63.3	57.2
	eGFP	p35S	27.0	-
pN2XJ48	COX4-TS-NifB$^{\textit{Ruminococcus albus SY3}}$	pE35S	39.2	35.7
	eGFP	p35S	27.0	-
pN2XJ49	SSU-TS-NifB$^{\textit{Ruminococcus albus SY3}}$	pE35S	41.8	35.7
	eGFP	p35S	27.0	-
pN2XJ50	COX4-TS-NifB$^{\textit{Paenibacillus sabinae T27}}$	pE35S	57.2	53.7
	eGFP	p35S	27.0	-
pN2XJ51	SSU-TS-NifB$^{\textit{Paenibacillus sabinae T27}}$	pE35S	59.8	53.7
	eGFP	p35S	27.0	-
pN2XJ52	COX4-TS-NifB$^{\textit{Syntrophobacter fumaroxidans MPOB}}$	pE35S	52.5	49.0
	eGFP	p35S	27.0	-
pN2XJ53	SSU-TS-NifB$^{\textit{Syntrophobacter fumaroxidans MPOB}}$	pE35S	55.1	49.0
	eGFP	p35S	27.0	-

pN2XJ54	COX4-TS-NifNB*Clostridium pasteurianum BC1*	pE35S	109.8	106.3
	eGFP	p35S	27.0	-
pN2XJ55	SSU-TS-NifNB*Clostridium pasteurianum BC1*	pE35S	112.4	106.3
	eGFP	p35S	27.0	-
pN2XJ56	COX4-TS-NifB*Rhodopseudomonas palustris CGA009*	pE35S	63.1	59.6
	eGFP	p35S	27.0	-
pN2XJ57	SSU-TS-NifB*Rhodopseudomonas palustris CGA009*	pE35S	65.7	59.6
	eGFP	p35S	27.0	-
pN2XJ58	COX4-TS-NifB*Desulfovibrio vulgaris DSM19637*	pE35S	40.2	36.7
	eGFP	p35S	27.0	-
pN2XJ59	SSU-TS-NifB*Desulfovibrio vulgaris DSM19637*	pE35S	42.8	36.7
	eGFP	p35S	27.0	-
pN2XJ60	COX4-TS-NifB*Chlorobium tepidum DSM 12025*	pE35S	53.5	50.0
	eGFP	p35S	27.0	-
pN2XJ61	SSU-TS-NifB*Chlorobium tepidum DSM 12025*	pE35S	56.1	50.0
	eGFP	p35S	27.0	-
pN2XJ62	COX4-TS-NifB*Methanosarcina acetivorans DSM 2834*	pE35S	42.9	39.4
	eGFP	p35S	27.0	-
pN2XJ63	SSU-TS-NifB*Methanosarcina acetivorans DSM 2834*	pE35S	45.5	39.4
	eGFP	p35S	27.0	-
pN2XJ64	COX4-TS-NifB*Methanothermobacter thermautotrophicus DSM 1053*	pE35S	39.2	35.7
	eGFP	p35S	27.0	-
pN2XJ65	SSU-TS-NifB*Methanothermobacter thermautotrophicus DSM 1053*	pE35S	41.8	35.7
	eGFP	p35S	27.0	-
pN2XJ66	COX4-TS-NifB*Geobacter metallireducens GS-15*	pE35S	38.3	34.8
	eGFP	p35S	27.0	-
pN2XJ67	SSU-TS-NifB*Geobacter metallireducens GS-15*	pE35S	40.9	34.8
	eGFP	p35S	27.0	-
pN2XJ68	COX4-TS-NifB*Synechococcus sp. JA-3-3A*	pE35S	60.1	56.6
	eGFP	p35S	27.0	-
pN2XJ69	SSU-TS-NifB*Synechococcus sp. JA-3-3A*	pE35S	62.7	56.6
	eGFP	p35S	27.0	-
pN2XJ70	COX4-TS-NifB*Nostoc azollae strain 0708*	pE35S	59.8	56.3
	eGFP	p35S	27.0	-
pN2XJ71	SSU-TS-NifB*Nostoc azollae strain 0708*	pE35S	62.4	56.3
	eGFP	p35S	27.0	-
pN2XJ72	COX4-TS-NifB*Cyanothece sp. PCC 7425*	pE35S	61.9	58.4
	eGFP	p35S	27.0	-
pN2XJ73	SSU-TS- NifB*Cyanothece sp. PCC 7425*	pE35S	64.5	58.4
	eGFP	p35S	27.0	-
pN2XJ74	COX4-TS-NifB*Rhodobacter capsulatus SB 1003*	pE35S	60.4	56.9
	eGFP	p35S	27.0	-
pN2XJ75	SSU-TS-NifB*Rhodobacter capsulatus SB 1003*	pE35S	63.0	56.9
	eGFP	p35S	27.0	-

pN2XJ76	COX4-TS-NifNB$^{\text{Methanosarcina acetivorans DSM 2834}}$	pE35S	101.7	98.2
	eGFP	p35S	27.0	-
pN2XJ77	SSU-TS-NifNB$^{\text{Methanosarcina acetivorans DSM 2834}}$	pE35S	104.3	98.2
	eGFP	p35S	27.0	-
pAE534 (MoClo level 2 plasmid)	COX4-TS-NifB^{Mi}	2x CaMV p35 + 5’UTR TMV	41.7	38.2
	SU9-FdxN^{Av}-HA	p35S	18.0	10.7
	SU9-NifU^{Av}	p35S	40.8	33.6
	SU9-NifS^{Av}	p35S	51.3	44.0
	eGFP	p35S	27.0	-
	p19	p35S	19.4	-
pAE535 (MoClo level 2 plasmid)	COX4-TS-NifB^{Mt}	2x CaMV p35 + 5’UTR TMV	39.2	35.7
	SU9-FdxN^{Av}-HA	p35S	18.0	10.7
	SU9-NifU^{Av}	p35S	40.8	33.6
	SU9-NifS^{Av}	p35S	51.3	44.0
	eGFP	p35S	27.0	-
	p19	p35S	19.4	-
pAE565 (MoClo level 2 plasmid)	SSU-TS-NifB^{Mi}	2x CaMV p35 + 5’UTR TMV	44.3	38.2
	SSU-FdxN^{Av}-HA	p35S	16.8	10.7
	SSU-NifU^{Av}	p35S	39.7	33.6
	SSU-NifS^{Av}	p35S	50.1	44.0
	eGFP	p35S	27.0	-
	p19	p35S	19.4	-
pAE569 (MoClo level 2 plasmid)	SSU-TS-NifB^{Ma}	2x CaMV p35 + 5’UTR TMV	45.5	39.4
	SSU-FdxN^{Av}-HA	p35S	16.8	10.7
	SSU-NifU^{Av}	p35S	39.7	33.6
	SSU-NifS^{Av}	p35S	50.1	44.0
	eGFP	p35S	27.0	-
	p19	p35S	19.4	-

FL, full-length; P, processed by removal of mitochondria or chloroplast targeting peptide.
