# Supplementary material for: Functional Nitrogenase Cofactor Maturase NifB in Mitochondria and Chloroplasts of Nicotiana benthamiana
Source: mBio. 2022 Jun 13;13(3):e00268-22. doi: 10.1128/mbio.00268-22 (PMC9239050; doi:10.1128/mbio.00268-22)
Supplement: TABLE S4 [file mbio.00268-22-s0005.pdf]

Table S4. List of modular pieces and primers used for the generation of transcriptional units to assemble multigenic vectors using MoClo cloning.

**Modular piece (Level 0)**	**Amplification Primers (5' – 3') (Mutagenesis primers)**	**Template**
nifB^{Mi}	ATGAAGACATCTCATTCGATGGAAAAGATGTCCAAGTTCTC<br>ATGAAGACATCTCGAAGCTCAATGAGAGAAGTGCTTCA	pN2XJ21
nifB^{Mt}	ATGAAGACATCTCATTCGATGCCAGATCAAAGACAAAC<br>ATGAAGACATCTCGAAGCTTAGTAATGAGAAGCTGGGG	pN2XJ64
nifB^{Ma}	ATGAAGACATCTCATTCGATGCCAGAAGAAAACCAGCC<br>ATGAAGACATCTCGAAGCTTAAGCTTCTTTCTTACCGT	pN2XJ63
fdxn^{Av}-ha	ATGAAGACATCTCAAGCCATGGCTCTTAAGATAGTTGA<br>ATGAAGACATCTCGAAGCTTAAGCATAATCTGGAACA	pN2XJ63
nifS^{Av}	-	(1)
nifU^{Av}	-	(1)
TwinStrep	-	(1)
CaMV p35S	-	pICH51266 (2)
2x CaMV p35s + 5'UTR TMV	-	pICH51288 (2)
pNOS+Ω	-	pICH87633 (2)
ssu (RcbS)	-	(1)
cox4	-	(3)
su9	-	(3)
eGFP	-	pICH41531 (2)
p19	-	pICH44022 (2)
T35s	-	pICH41414 (2)
tNOS	-	pICH41421 (2)

Transcriptional Units (level 1)	Modular pieces used	Entry vector
cox4-ts-nifB^{Mi}	2xp35S::*cox4-ts-nifB^{Mi}*-tNOS	pICH47732
cox4-ts-nifB^{Mt}	2xp35S::*cox4-ts-nifB^{Mt}*-tNOS	pICH47732
su9-fdxn^{Av}-HA	p35S::*su9-fdxn^{Av}-ha*-T35s	pICH47742
su9-nifU^{Av}	p35S::*su9-nifU^{Av}*-T35s	pICH47761
su9-nifS^{Av}	p35S::*su9-nifS^{Av}*-T35s	pICH47772
ssu-ts-nifB^{Mi}	2xp35S::*ssu-ts-nifB^{Mi}*-tNOS	pICH47732
ssu-ts-nifB^{Ma}	2xp35S::*ssu-ts-nifB^{Ma}*-tNOS	pICH47732
ssu-fdxn^{Av}-ha	p35S::*ssu-fdxn^{Av}-ha*-T35s	pICH47742

ssu-nifU^{Av}	p35S::*ssu-nifU^{Av}*-T35s	pICH47761
ssu-nifS^{Av}	p35S::*ssu-nifS^{Av}*-T35s	pICH47772
p19	pNOS+Ω::*p19*-tNOS	pICH47751
cytoplasmic *eGFP*	p35S::*eGFP*-T35s	pICH47781

References

1. Eseverri A, Lopez-Torrejon G, Jiang X, Buren S, Rubio LM, Caro E. 2020. Use of synthetic biology tools to optimize the production of active nitrogenase Fe protein in chloroplasts of tobacco leaf cells. Plant Biotechnol J 18:1882-1896.
2. Engler C, Youles M, Gruetzner R, Ehnert T-M, Werner S, Jones JDG, Patron NJ, Marillonnet S. 2014. A Golden Gate modular cloning toolbox for plants. ACS SynthBiol 3:839-843.
3. Jiang X, Paya-Tormo L, Coroian D, Garcia-Rubio I, Castellanos-Rueda R, Eseverri A, Lopez-Torrejon G, Buren S, Rubio LM. 2021. Exploiting genetic diversity and gene synthesis to identify superior nitrogenase NifH protein variants to engineer N_2-fixation in plants. Commun Biol 4:4.
